# Chemical approaches for the enhancement of porphyrin skeleton-based photodynamic therapy

**DOI:** 10.1080/14756366.2020.1755669

**Published:** 2020-04-24

**Authors:** Yuyan Lin, Tao Zhou, Renren Bai, Yuanyuan Xie

**Affiliations:** a Collaborative Innovation Centre of Yangtze River Delta Region Green Pharmaceuticals, Zhejiang University of Technology, Hangzhou, China; b School of Food Science and Biotechnology, Zhejiang Gongshang University, Hangzhou, China; c College of Pharmaceutical Science, Zhejiang University of Technology, Hangzhou, China

**Keywords:** PDT, PS, porphyrin, photoactivity, anti-cancer

## Abstract

With the development of photodynamic therapy (PDT), remarkable studies have been conducted to generate photosensitisers (PSs), especially porphyrin PSs. A variety of chemical modifications of the porphyrin skeleton have been introduced to improve cellular delivery, stability, and selectivity for cancerous tissues. This review aims to highlight the developments in porphyrin-based structural modifications, with a specific emphasis on the role of PDT in anticancer treatment and the design of PSs to achieve a synergistic effect on multiple targets.

## 1. Introduction

**Figure 1.**
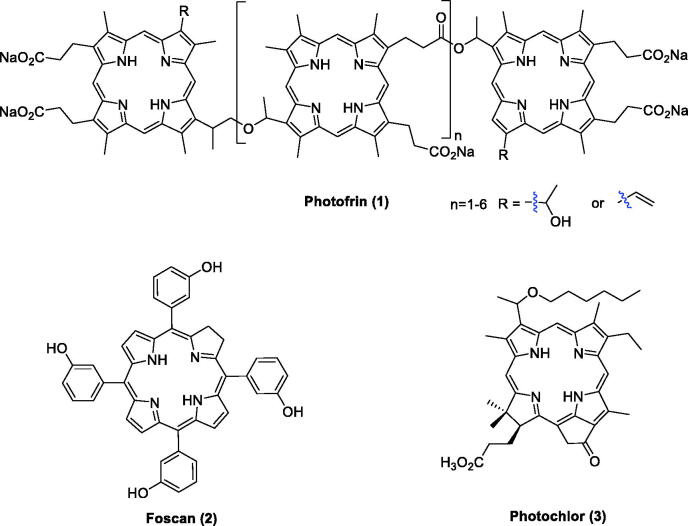
PSs that entered clinical trials.

As the population ages, the number of cancer cases and deaths worldwide is also rapidly growing^1^^,^^2^. With the continuous development of medicine, the treatment strategies for cancer are also constantly improving. Photodynamic therapy (PDT) has been considered a safer cancer therapy approach with fewer side effects^3^. In 1978, Dougherty first applied this technique to gastrointestinal cancer using hematoporphyrin (HPD)^4^^,^^5^. Clinical studies revealed that PDT has been increasingly utilised in therapy for solid tumours, including tumours of the brain, head and neck, skin, oesophagus, lung, gastrointestinal, bone, bladder, prostate, breast, cervix, and ovary and in basal cell carcinomas^6^^,^^7^. Porfimer sodium (Photofrin^®^, Figure 1) was the first photosensitiser (PS) approved worldwide for the treatment of cancer. It has no long-term side effects and can be used repeatedly without causing drug resistance^8^. As an effective combination therapeutic strategy, Photofrin^®^ did not display serious toxicity. Moreover, the survival period of inoperable tumour patients was prolonged, and the quality of life improved^9^^,^^10^. However, patients still suffered several side effects during the treatment, including skin photosensitivity and metabolic disturbances^10^.

### 1.1. Mechanism of PDT

PSs usually accumulate in the tumours of mice after intravenous injection. After irradiation with a red laser beam at a specific wavelength of light, photodynamic therapy is activated by the absorption of a photon, followed by the oxidation and degradation of vital biomolecules^3^.

**Figure 2.**
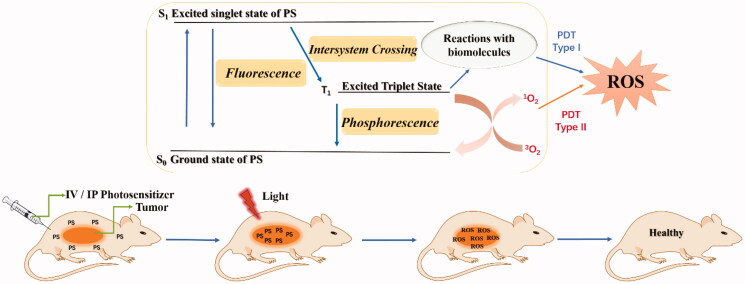
Photosensitisation process and mechanism of action of PDT.

As illustrated in Figure 2, the anti-tumour mechanism of PDT mainly consists of two stages. In the first stage, after the absorption of light, the PS transforms from the ground singlet state (S_0_) into the singlet excited state (S_1_) (nanosecond range), followed by conversion to the excited triplet state T_1_ (micro to millisecond range). In the excited triplet state, the PS can undergo two types of reactions (type I or type II reactions). In the type I pathway, an electron or hydrogen atom transfer occurs between the triplet state T_1_ sensitiser and the cell membranes of biomolecules. This process forms free radicals and radical ions, leading to the generation of cytotoxic hydroxyl radicals (^•^OH), hydrogen peroxides (H_2_O_2_) and other reactive oxygen species (ROS). The type II reaction involves the interaction between the electronically excited triplet sensitiser and triplet ground-state molecular oxygen (^3^O_2_). ^3^O_2_ then forms singlet oxygen (^1^O_2_) using the energy transferred from the excited PS. Through its reactions with many biological molecules, the product ^1^O_2_ is the key factor that induces apoptosis of cancer cells and tissue destruction. Moreover, although the type II reaction has been confirmed to play a more important role in PDT, both the type I and type II reactions can occur independently at the same time^10–12^.

### 1.2. Advantage of porphyrin PSs

Most of the PSs applied to cancer treatment possess a macrocyclic framework based on the porphyrin skeleton^13^. The main advantages of porphyrins as PSs in PDT include 1) aromatic stability; 2) efficient absorption of visible red light; 3) high yield of active oxygen; 4) easily functional modification and structural diversity; and 5) long triplet state lifetime and minimal dark toxicity^3^^,^^12^^,^^14^. Several PSs, such as Photofrin^®^, 5,10,15,20-tetrakis(3-hydroxyphenyl)chlorin (Foscan^®^), and 3–(1-hexyloxyethyl)-3-divinylpyropheophorbide (Photochlor^®^)^15^, have been approved for the treatment of various cancers (Figure 1)^16^^,^^17^.

Obviously, any potential PS agent used in PDT should meet the following requirements: 1) should be a single compound with tuneable amphiphilicity, high purity and satisfying yield through mature synthetic methodology^10^^,^^16^; 2) have acceptable dark toxicity; 3) induce high yields of ^1^O_2_ and reasonable fluorescence quantum yields; 4) have high tumour selectivity and exhibit rapid accumulation and long retention targeting of tumour tissues^18^; 5) should efficiently absorb red or far-red light to penetrate tissues; 6) show no excessive aggregation in biological environments resulting in a reduction in its photochemical efficiency; and 7) exhibit rapid pharmacokinetic elimination from the patient^11^^,^^19^.

## 2. Structural optimisation based on porphyrin skeletons

To meet the above requirements, it is important to discover and develop more effective ideal PSs. For example, one of the possible approaches to avoid porphyrin aggregation is the construction of organised porphyrin systems, such as metal-organic frameworks with porphyrin linkers^20^^,^^21^. Additionally, the insertion of halogen atoms increases the intersystem crossing quantum yield and leads to the generation of high ROS yields^22^. Another strategy is to improve the specificity of a PS to minimise its toxicity and adverse effects. In addition, appropriate substituents at appropriate positions in the porphyrin can favourably influence the lipophilicity and tissue distribution of PSs^12^^,^^19^.

**Figure 3.**
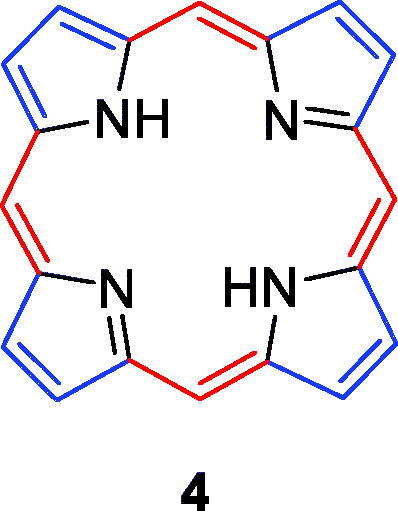
Backbone chemical structure of porphyrin.

In this manuscript, we review the latest research progress in porphyrin-based structural modifications designed to increase photocytotoxicity and selective accumulation in the tumour. The modifications are mainly performed at three positions or moieties, the *meso*-position (red), the *β*-pyrrolic position (blue) and the hydrogen bonding/metal coordination moiety (black; Figure 3). We are looking forward to providing useful information to researchers for the design and synthesis of more excellent porphyrin-based PSs.

### 2.1. Functional group-modified porphyrin derivatives

**Figure 4.**
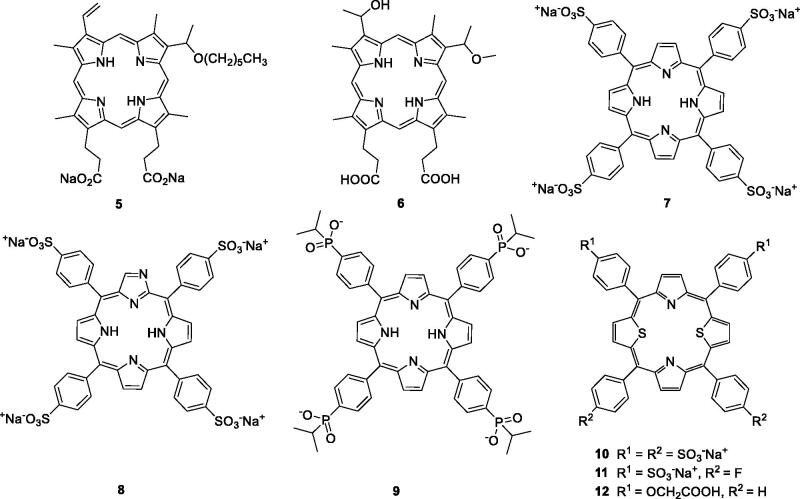
Structures of porphyrin conjugates **5–12**.

Structural differences among porphyrin isomers have a great impact on photodynamic activity. Feng et al.^16^ prepared and evaluated a novel series of water-soluble porphyrin derivatives containing carboxyl and *n*-hexyl groups. The results indicated that compound **5** exhibited high dark toxicity/phototoxicity ratios in MDA-MB-231 cells (compound **5 **=** **160.88) compared with hematoporphyrin monomethyl ether (HMME, **6 **=** **18.76). Compound **7** was localised at higher absolute concentrations in some tumours, but its neurotoxicity resulted in failure of its development as a PS^23^^,^^24^. To avoid the neurotoxicity associated with compound **7**, a series of its derivatives were investigated (Figure 4)**.** Thomas et al.^25^ designed and prepared compound **8**, a water-soluble derivative of *N*-fused porphyrin (NCP), displaying an IC_50_ value of 6 µM (irradiation with 100 J/cm^2^ of a 70 W sodium vapour lamp) against MDA-MB-231 cells.

Recently, Hynek et al.^26^ first synthesised porphyrin derivatives containing four phosphinic functional groups with methyl, isopropyl and phenyl groups on phosphorus atoms. These compounds were evaluated for their *in vitro* anticancer activity against the HeLa cell line. It was shown that the presence of phosphinic groups did not influence the photophysical properties and absorption. Derivative **9**, with four isopropyl substituents, exerted promising anticancer activity, with a mean IC_50_ value of 0.45 µM (irradiation with 9 mW/cm^2^ of 525 nm light for 15 min), which was 6-fold more potent than compound **7** (Figure 4).

Structural modification of heteroatoms by introducing chalcogen atoms, such as sulphur and selenium, significantly changes the photophysical properties of compounds^27^. The incorporation of ionic groups such as pyridinium, sulphonate, carboxylate or phosphonate around the porphyrin is an effective method to address solubility issues and prevent aggregations^26^.

In 2000, Stilts et al.^28^ synthesised water-soluble core-modified porphyrins (**10–12**). The LD_50_ of compound **10** was less than 23.6 µM against Colo-26 cells (irradiation with 135 J/cm^2^ of 694 nm). In their subsequent work, studies on the *in vivo* application of dithiaporphyrin **11** demonstrated that it could absorb light at much longer wavelengths compared with compound **1**, resulting in an enhanced penetration depth of light, more potent dark toxicity and more accurate tumour localisation. The EC_50_ value of compound **11** was 1.6 µM (irradiation with 4 J/cm^2^ of 590–800 nm light), much favourable than that of compounds **1** (EC_50_ = 9.0 µM) and **7** (EC_50_ = 125 µM)^29^^,^^30^. Based on the influence of the p*K*a value and previous studies, core-modified porphyrins **12** were obtained by using a carboxylic acid group instead of a sulphonic acid group. Studies revealed that the introduction of carboxyl groups into the porphyrin ring led to an increase in the phototoxicity compared with the corresponding sulphonic acid group (compound **11**), but porphyrin derivatives with more than three carboxylic acid displayed essentially no phototoxicity, which correlates with greatly reduced cellular uptake^27^.

**Figure 5.**
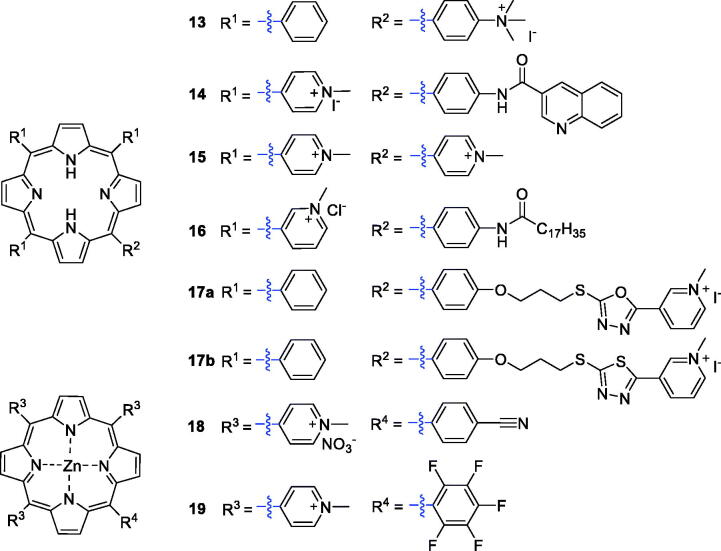
Structures of cationic porphyrin derivatives **13–19**.

Cationic porphyrin derivatives have received special attention because of their potential interaction with anionic DNA/RNA and efficient cell destruction upon irradiation^31^. Slomp’s group synthesised a series of cationic porphyrin derivatives and screened their photosensitising activities against HaCaT keratinocytes. The results confirmed that cationic porphyrin PSs exhibited better photosensitivity than derivatives with neutral or negatively charged substituents^32^. Jensen et al.^33^ then synthesised several cationic porphyrin derivatives possessing –(CH_3_)_3_^+^ groups. The monocationic porphyrin derivative **13** (Figure 5), with only one cationic group, was found to be the most active against HEp2 cells (IC_50_ = 2 µM, irradiation with 1 J/cm^2^ of 610 nm light). Researchers also reported a cationic aminoporphyrin-quinoxaline hybrid (**14**), formed by introducing a quinoxaline carboxylic acid group, that exhibited potential anticancer activity. All the compounds synthesised exhibited 5-HT3 receptor antagonism, and some showed antagonism greater than the reference drug. Hybrid **14** displayed an IC_50_ value of 0.06 µM (under a white LED light source, *λ* = 400–800 nm, 2 mW and irradiation for 10 min.) against the A549 cancer cell line, showing 5-fold better inhibitory activity than the reference compound **15** (H_2_TMPyP, IC_50_ = 0.30 µM)^34^.

Jelovica et al.^35^ designed and synthesised an amphiphilic porphyrin derivative with a long lipophilic alkyl side chain. The solubility of the compound was markedly improved after the introduction of three 3-pyridyl groups. Compound **16** showed the most potent phototoxicity against HeLa and u87MG cell lines, with IC_50_ values of 0.11 and 0.15 µM, respectively (irradiation with 3.6 J/cm^2^ of 630 nm). Oxazole, 1,3,4-oxadiazole and 1,3,4-thiadiazole structures were reported to exert biological effects in inhibiting tumour vessel growth^36^. Zheng et al.^37^ synthesised a series of porphyrin derivatives with the above pharmacophores, and compounds **17a–b** displayed moderate ^1^O_2_ yielding and DNA photocleavage activities (Figure 5).

Cationic porphyrins have a high DNA binding/photolysis potential, and the presence of central metal ions plays a key role in forming complexes with DNA^38^^,^^39^. Antoni et al.^40^ reported the synthesis and biological evaluation of Zn-porphyrins (**18**), which showed high cytotoxicity towards the A2780 human cancer cell line, with IC_50_ values as low as 0.4 µM when irradiated with red light at a wavelength greater than 600 nm. Then, Yoho et al.^41^ synthesised a new series of water-soluble zinc(II) porphyrin derivatives structurally related to a pentafluorophenyl *meso*-substituent, which were assessed for anticancer activity against non-small cell lung cancer (NSCLC). *In vitro* and *in vivo* results indicated that **19** displayed high phototoxicity and low dark toxicity against NSCLC. It is worth noting that **19** caused apoptosis of NSCLC cells at a concentration as low as 75 nM when irradiated with 420 nm light at a power density of 2.3 W/cm^2^. These results indicate that derivative **19** could be considered as a lead in development of potential effective mono-therapeutic agents for treatment of lung cancer (Figure 5).

**Figure 6.**
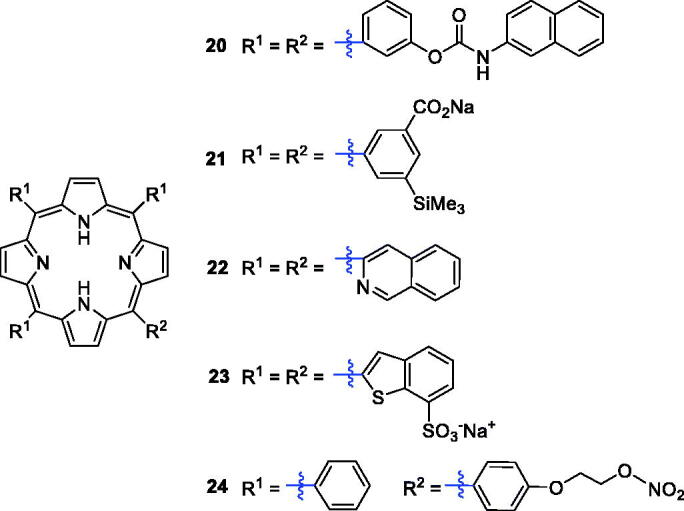
Structures of porphyrin conjugates **20–24**.

Previous studies have shown that naphthyl-isocyanate can participate in the ROS formation and exhibits significant *in vitro* toxic effects on tumour cell lines^42^^,^^43^. Compound **20** (Figure 6), possessing an amide-naphthyl moiety, was synthesised and its photodynamic activities were evaluated against HT-29 cells. It showed low dark cytotoxicity and the best *in vitro* activity (IC50 = 4.848 µM) when incubated with light between 600 and 800 nm^44^. Additionally, silicon is considered to have potential for improving photochemical efficiencies and has been introduced as a metal centre in phthalocyanine, and the results have shown it to be a promising PS for PDT^45^^,^^46^. Horiuchi et al.^47^ introduced silyl groups into the ring of tetraphenylporphyrin to obtain compound **21** (Figure 6), which showed high selective accumulation efficiency (the concentration of compound **21** was 13-fold higher than that in muscle 12 h after drug administration) in tumours. Derivative **22** (Figure 6) attached to a quinoline group showed various pharmaceutical activities, including anti-tumour activity, and meets several essential requirements of an ideal PS; for example, it produced ^1^O_2_ efficiently (Φ_Δ_ = 0.62) in tetrahydrofuran. The calculated value was above the range reported for most PSs employed in PDT^48^^,^^49^. Benzothiophene-containing porphyrin derivatives were found to selectively accumulate in the mitochondria and nucleus of MCF-7 cells. Rangasamye et al.^50^ also reported a novel compound (**23**) displaying a low dark cytotoxic effect, and under light conditions (660 nm, 50 mW, 30 min), it showed more effective activity (IC_50_ = 5.0 µM) than compound **7** (IC_50_ = 11.76 µM).

Recent evidence from clinical studies indicates that high concentrations of NO have certain cytotoxicity, can induce tumour cell apoptosis, and help macrophages kill tumour cells^51^. Thus, a series of nitrates NO-donor porphyrin derivatives were prepared to enhance anti-tumour activity (Figure 6)**.** Compound **24** exerted the most potent activity against MCF-7 breast cancer cells (IC_50_ = 0.8 µM), which was much better than the reference drug 5-fluorouracil at a wavelength of 570 nm (IC_50_ = 4.3 µM)^52^.

Li et al.^53^ investigated a novel porphyrin derivative **25** (Figure 9) that exhibited favourable anti-tumour toxicity both *in vitro* and *in vivo.* A series of compounds was obtained by modification of tetraarylporphyrin rings, and the 4-OH-phenyl derivative (**26**, Figure 9, IC_50_ = 3.07 ng/mL or 4.52 nM and irradiation with halogen lamp 500 W for 2 h light irradiance 5.5 × 10^−2^ mW/cm^2^·n m) was significantly more potent than compound **1** (IC_50_ = 73.67 ng/mL)^54^.

**Figure 7.**
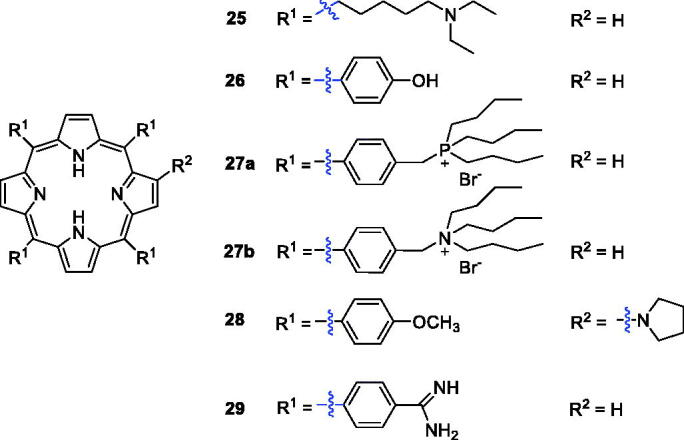
Structures of porphyrin conjugates **25–29**.

Hudson et al.^55^ synthesised phosphorous (**27a**) and nitrogen (**27 b**) centred lipophilic cationic porphyrins displaying LD_90_ values of 5.9 and 6.1 µM (irradiation with 3.6 J/cm^2^ of 630 nm light), respectively, during *in vitro* photodynamic assays against human colorectal adenocarcinoma cells (HT-29). Liao et al.^56^ reported hybrids of *β*-alkylaminoporphyrins and different amines or substituted phenyl groups. Compound **28** showed better phototoxicity against HeLa cells (IC_50_ = 4.38 µM, 650 nm, 16 J/cm^2^). Li’s^57^ group prepared 5,10,15,20-tetrakis(4-amidinophenyl)porphyrin **29** (Figure 7), which produced singlet oxygen more efficiently and displayed binding activity and photodamage to DNA and tumour cells. In 2015, the same research group reported that the cytotoxicity of derivative **29** was 90% at 4 µM and 12 J/cm^2^ ^58^.

### 2.2. Biomolecule-conjugated porphyrin derivatives

**Figure 8.**
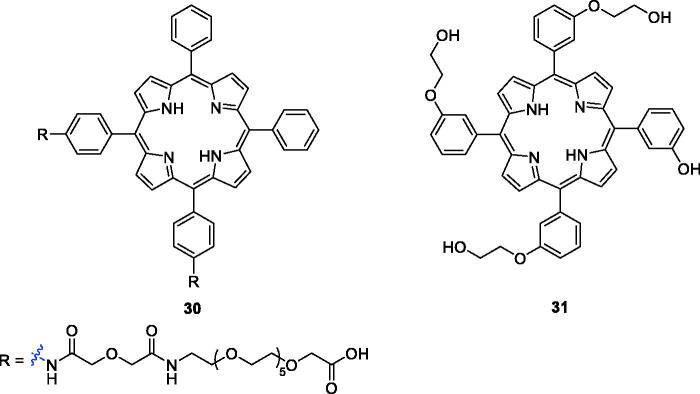
Structures of porphyrin conjugates **30** and **31**.

Polyethylene glycol (PEG) is frequently applied as common covalent linker in drug delivery strategies^59^. A new series of PEG-functionalised porphyrins were synthesised and screened for biological activity against human HEp2 cells. The hydrophobicity of the PEG-porphyrins decreased when the number of PEG chains attached to the porphyrin ring increased. None of the PEG-porphyrins had dark toxicity, and derivative **30** (Figure 8) was the most potent compound, with an IC_50_ value of 1.8 µM (1 J/cm^2^, exposed to light from a 100 W halogen lamp)^60^.

Králová et al.^61^ designed and synthesised glycol-functionalised porphyrins that were linked to the phenyl group of *meta*-tetraphenylporphyrin via ether bonds and incorporation of fluorine. Compared with compound **2**, which also exhibited phototoxicity against HL60 (IC_50_ = 42.0 nM at 13.3 J/cm^2^, 620–660 nm) and 4T1 (IC_50_ = 117.0 nM at 13.3 J/cm^2^, 620–660 nm) cells, derivative **31** showed the highest phototoxicity against HL60 (IC_50_ = 31.0 nM at 2.5 J/cm^2^, 500–520 nm) and 4T1 (IC_50_ = 93.0 nM at 2.5 J/cm^2^, 500–520 nm) cells. The results had important guiding significance in PDT.

**Figure 9.**
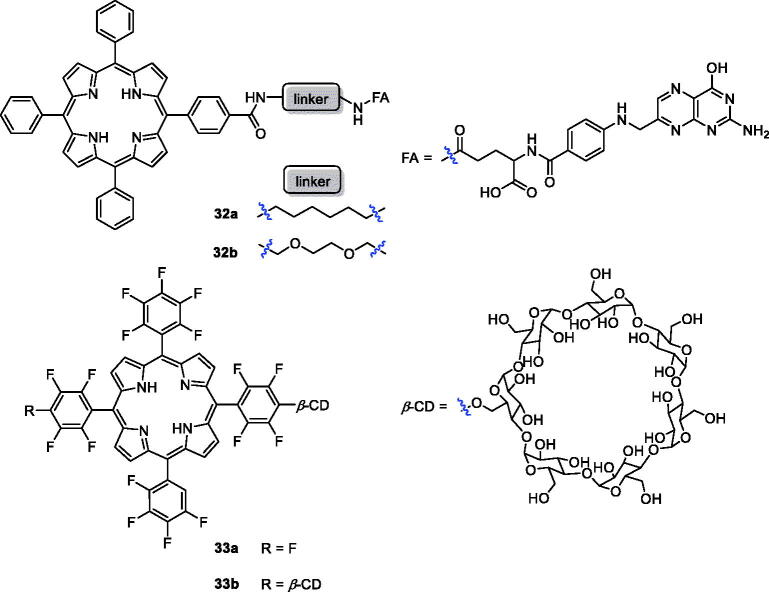
Structures of porphyrin conjugates **32** and **33**.

Among the different strategies for developing receptor-mediated delivery systems, folate receptor (FR) is a useful target for tumour-specific drug delivery for the following reasons: (1) it is upregulated in many human cancers; (2) the density of folate receptor increases as cancer progresses; (3) folate has high affinity for FRs on the cell surface^62^. Two new conjugates of folic acid-porphyrin derivatives (**32a**–**b**) were synthesised and evaluated for biocompatibility and photodynamic activity against KB cells (Figure 9)**.** These compounds, with folate linked to a porphyrin ring, demonstrated 7 times more intracellular uptake than compound **1**. Under the same experimental conditions, the photodynamic activity of conjugate **32 b** was 3.4 times than that of **32a** (Cells were incubated with PSs at 10^−5 ^M for 24 h before light treatment. The LD_50_ values of **32a** and **32 b** were 22.6 and 6.7 J/cm^2^)^62^^,^^63^. Cyclodextrin is not only a drug carrier but can also improve physicochemical and pharmaceutical properties, such as solubility, stability, and bioavailability^64–67^. On the other hand, incorporation of fluorine into the porphyrin core can improve the pharmacodynamics and pharmacokinetic properties. Therefore, the authors’ strategy was to introduce one or two cyclodextrins into a porphyrin molecule and evaluate the antitumor activity of the products on mouse breast cancer 4T1 cells. The results indicate that **33 b** (EC_50_ =10 µM at 3.4 J/cm^−2^, 500–700 nm) was a novel photosensitising drug with selective tumour uptake and rapid tumour clearance^68^.

**Figure 10.**
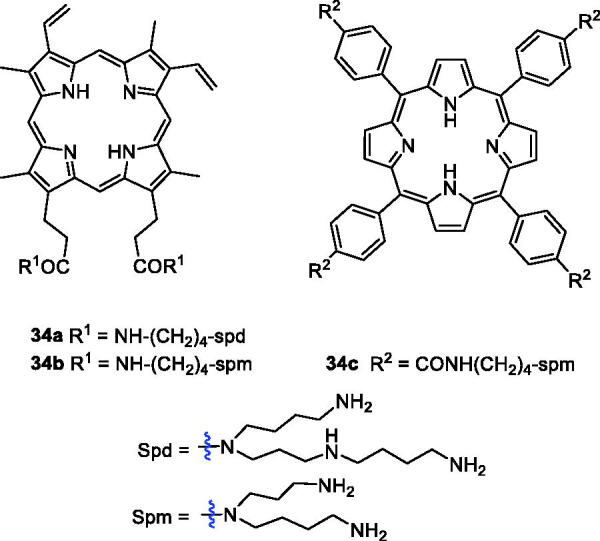
PA-conjugated porphyrin derivatives **34**.

The polyamine (PA) transport system can afford selective accumulation of PA analogues in neoplastic tissues and presents a very attractive anticancer chemotherapeutic strategy^69^. 5-Aminolaevulinic acid-based photodynamic therapy (5-ALA-PDT) received approval for cancer treatment in 1999. 5-ALA can produce protoporphyrin IX (PpIX), which exhibits a photosensitising property. Sol et al.^70^ revealed the potential of linking a PA to the porphyrin derivative and synthesised a new type of porphyrin PS by tethering polyamine moieties. Only PpIX polyamine derivatives **34a**–**b** were amphiphilic molecules, and PA porphyrin conjugate **34 b** exhibited good photocytotoxicity and remarkable selectivity against K562 human chronic myelogenous leukaemia cells, higher than that of compound **1** when irradiated with white light. Fidanzi-Dugas et al.^71^ synthesised PpIX derivatives containing a PA moiety in order to enhance tumour tissue targeting ability and analysed the effects of PDT on prostate cancer. The results showed that PpIX-PA was 7- to 14-fold more effective when vectorised with PA, and PpIX-PA had better efficacy than 5-ALA. Thus, Sarrazy et al.^72^ designed and synthesised a new porphyrin–PA conjugate **34c** by means of a flexible arm. Under the same experimental conditions (irradiation with visible light for 2 h and a fluence rate of 2.5 mW/cm^2^), the photodynamic activity of conjugate **32c** (IC_50_ = 49.4 µM) was 50 times greater than that of compound **1** against the MCF7 cell line. The better efficiency of porphyrin PA derivatives could be attributed to their more ready uptake vs compound **1** (Figure 10).

Attaching a glycosyl group to an anti-tumour drug allows the drug to target the tumour site and is an effective way to increase the water solubility of the drug^73^. One research group has reported that compounds with triglyco-conjugation were more photocytotoxic than the corresponding symmetrical tetra-glucoconjugated compounds^74^. The photodynamic activity of the tri-glucoconjugated porphyrin 5,10,15-mesotri-(*meta*-*O*-*β*-d-glucosyloxyphenyl)-20-phenylporphyrin [*m*-TPP(glu)_3_] (**35**) was investigated and compared with that of non-glucoconjugated compound **2** by Desroches et al.^75^ The results of an *in vivo* animal experiment showed that compound **35** was more attractive than compound **2** as a PS. Further analysis verified that the photosensitivity of the skin may be prolonged by slow elimination, while compound **35** can avoid this.

**Figure 11.**
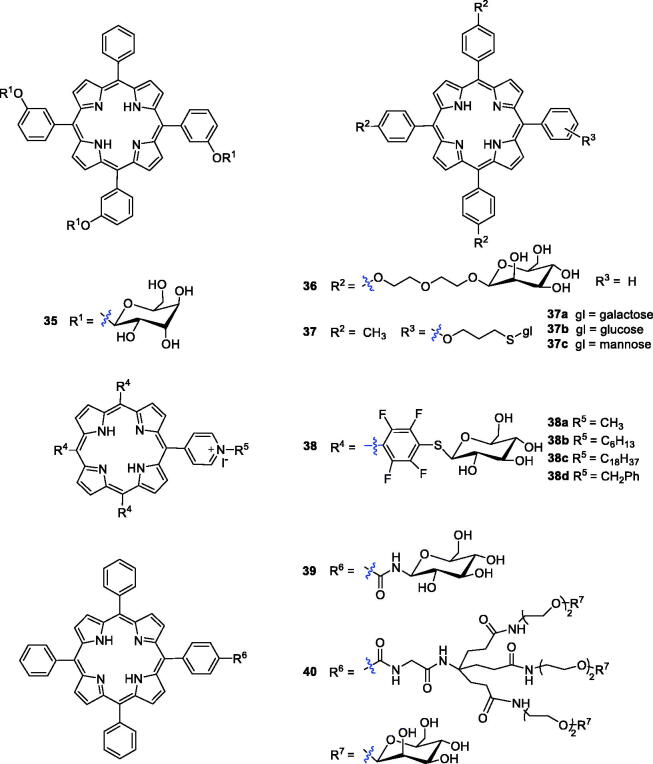
Glycoconjugated porphyrin derivatives **35–40**.

Glycol-conjugated porphyrins were synthesised using diethylene glycol (Deg)-linked O- and S-galacto/manno-conjugated *m*-tetraphenyl porphyrins and were evaluated for photobiological activity in colorectal adenocarcinoma (HT29) and retinoblastoma (Y79) cell lines (Figure 11). Compound **36** with mannose showed good cytotoxicity against the Y79 cell line, with an IC_50_ value of 0.35 µM after illumination with red light at 1.8 J/cm^2^, which was much better than compound **35** (IC_50_ = 1.9 µM)^76^^,^^77^.

In comparison with O-glycosylated porphyrins, *S*-glycosyl bonds and –CONH– glycosyl bonds can resist endogenous hydrolysis catalysed by glycosidases^78^. To identify novel porphyrin analogues with PS candidate potential, one research group reported the synthesis of a new family of glycosylated porphyrins in which the sugar moieties (glucose, mannose and galactose) were linked to the tetrapyrrole ring by a thioglycosidic bond. The initial results revealed that all the *ortho* isomers (**37a**–**c**) had photodynamic activity (Figure 11)^79^. Kaldapa et al.^80^ synthesised a porphyrin derivative having both a sugar-containing residue and a positive-charge group as a PDT sensitiser, which exhibited better water solubility and greater membrane permeability than porphyrins solubilised solely by multiple charged groups.

Ahmed et al.^81^ reported *meso*-tetraaryl porphyrins substituted with three thioglycosyl units and one pyridyl substituent simultaneously, and their photodynamic activity was screened against human colorectal adenocarcinoma cells (HT-29). Four glycosyl cationic porphyrins were able to kill 90% of the cells in the micromolar range. Among these four compounds, **38c** showed the best phototoxicity (LD_90_ = 25 µM), and **38d** was the least active (LD_90_ = 50 µM), while **38a**–**b** exhibited intermediate activity of 35 µM and 45 µM, respectively, when irradiated with cooled filtered red light (630 nm, 3.6 J/cm^2^). All the compounds showed negligible dark toxicity towards the cells at the highest concentration. Stasio et al.^82^ synthesised mono-and di-glucosylated porphyrins and compared their photocytotoxic properties against HT29 human adenocarcinoma cells with those of tetraphenylporphyrin. The cellular uptake and LD_50_ values of compound **39** were approximately 11.5- and 2.7-fold higher than those of tetraphenylporphyrin (cells were incubated with photosensitisers at 10^−6 ^M for 24 h The light doses yielding 50% growth inhibition (LD_50_) values of **39** and TPP were 11.1 and 30.2 J/cm^−2^ at 650 nm).

Other drug carriers, such as dendrimers, have been explored as ideal delivery vehicle candidates for explicit study of the effects of polymer size, charge, composition, and architecture on biologically relevant properties^83^. Ballut et al.^84^ reported the synthesis of two symmetric dendrimers attached to a tetrasubstituted porphyrin via amide linkages. To increase photo efficiency, Ballut et al.^85^ designed and synthesised a new glycol-conjugated PS with only one glycodendrimer moiety, and the length between carbohydrate and porphyrin was variable. Biological evaluations showed that compound **40** was indeed embedded into the phospholipid bilayer and its sugar moieties protruded into the surrounding aqueous phase, again confirming that glycodendrimeric phenylporphyrin could be an efficient carrier for drug targeting in PDT. Griegel et al.^86^ demonstrated that human retinoblastoma cells overexpressed mannose and receptors, which significantly encouraged medicinal chemists to create PSs with enhanced targeting ability towards retinoblastoma cells. Compound **40** was compared with the non-dendrimeric tri-substituted derivative **36**. The phototoxicity of compound **40** (LD_50_ = 0.5 µM) in Y79 cells was observed to be the same order of magnitude as that of TPP(*p*-Deg-*O*-α-ManOH)_3_ **36** (LD_50_ = 0.7 µM after exposure to visible light at 1.2 J/cm^2^) Most importantly, the glycodendrimeric porphyrins possessed a lower cellular uptake and a higher affinity towards plasma proteins, making them possible candidates for PDT targeting the vasculature^87^.

Recently, Rosilio’s research group studied the influence of mannose residues and the geometry of porphyrin derivatives on cellular uptake and photodynamic effectiveness. The results showed that the phototoxic efficacy of a glycol-conjugated tetraarylporphyrin against retinoblastoma cells was not necessarily related to its interaction with a mannose receptor^88^. Among the derivatives, the porphyrin substituted by three diethylene glycol α-mannosyl groups (derivative **36**) was found to be the best PS candidate for PDT, and the glycosyl substituents in the porphyrin ring were found to have a strong relationship with dark toxicity. Generally, the combination of PSs and targeting systems is an ideal mode of administration, and targeted therapy systems will improve therapeutic capabilities against cancer.

### 2.3. Metal-modified porphyrin derivatives

**Figure 12.**
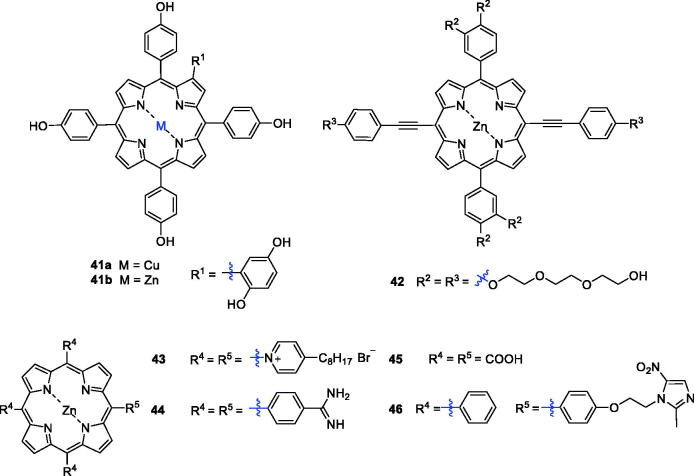
Porphyrin–zinc complexes **41–46**.

Metalloporphyrins are widely found in nature, and their ability to cleave DNA nucleases has drawn considerable attention over the last few years. Therefore, combining porphyrins with metals not only provides additional anti-tumour activity and tumour selectivity, but the biodistribution of the metal inside and outside the tumour cell can be tracked^89^. The PDT activity of metal complexes depends largely on the central metals due to the paramagnetic effect^90^. For the stability of the porphyrin ring and to maintain the photophysical properties, many researchers have added zinc into the porphyrin ring. In addition, the structure of *β*-substituted porphyrins is more similar to that of natural porphyrin than to *meso*-substituted porphyrins, and it is widely used in biological studies^91^. Huang et al.^92^ synthesised and characterised the novel PSs Zn(II) P (**41 b**) and Cu(II) P (**41a**). Compound **41 b** was found to induce necrosis or apoptosis of K562 human chronic myelogenous leukaemia cells under light irradiation, while **41a** exhibited inferior photosensitising activity (Figure 12).

Triethylene glycol moieties have a certain cell permeability, and zinc porphyrin has a better therapeutic effect than metal-free porphyrin. On this basis, a series of porphyrin PSs with a triethylene glycol moiety as a peripheral substituent was synthesised, and the products showed a strong absorption coefficient in the near-infrared region. These derivatives were assessed for activity against HeLa cells, and structure–activity relationship studies demonstrated that substitution of various moieties in the present porphyrins led to negligible dark toxicity and robust phototoxicity, especially for compound **42** (IC_50_ = 4.43 µM, after illumination with 650 nm at 40 mW/cm^2^ for 10 min) (Figure 12)^93^.

In 2009, Pavani et al.^94^ synthesised a series of *meso*-substituted tetra-cationic porphyrins PSs to study the effects of zinc on the membrane-binding property, cell subcellular localisation and cell phototoxicity. The results of this investigation indicated that the zinc derivative ZnTC8PyP (**43**) displayed the highest uptake. The presence of zinc reduced mitochondrial binding and promoted membrane binding due to its complexation with phospholipid phosphate groups, thereby increasing the efficiency of PDT. Thereafter, tetraphenylporphyrins with an amidine group were prepared^57^. Among them, Zn(II)-porphyrin **44** showed the greatest photocytotoxity, which could be attributed to its corresponding high triplet quantum yield of oxygen. However, the cytotoxicity of a bisporphyrin derivative against HK-1 cells was lower than that of compound **44**, likely due to the intermolecular aggregation of bisporphyrin rings that led to a decrease in singlet oxygen generation^58^.

Zhang et al.^90^ reported the design and preparation of Zn(II) 5,10,15,20-tetrakis(carboxyl)porphyrin (**45**). The photodynamic anticancer activity of compound **45** was investigated with MTT assays, using compound **2** as a positive control. Interestingly, after illumination (625 nm, 5 W, red light, 15.5 cm from the light source), compound **45** had better phototoxicity against A549 cells (IC_50_ = 16.0 µM), HeLa cells (IC_50_=46.3 µM) and HepG2 cancer cells (IC_50_ = 43.1 µM).

Yu et al.^95^ synthesised new metronidazole-appended porphyrins, which plays a significant role in biological metabolism, 5,10,15-tris (phenyl)-20-[4–(2-(2-methyl-5-nitro-imidazolyl)ethoxyl) phenyl]porphyrin H_2_Pp and its corresponding zinc(II) porphyrin ZnPp (**46**). Compound **46** exhibited nearly no cytotoxicity against breast cancer cells in the darkness.

**Figure 13.**
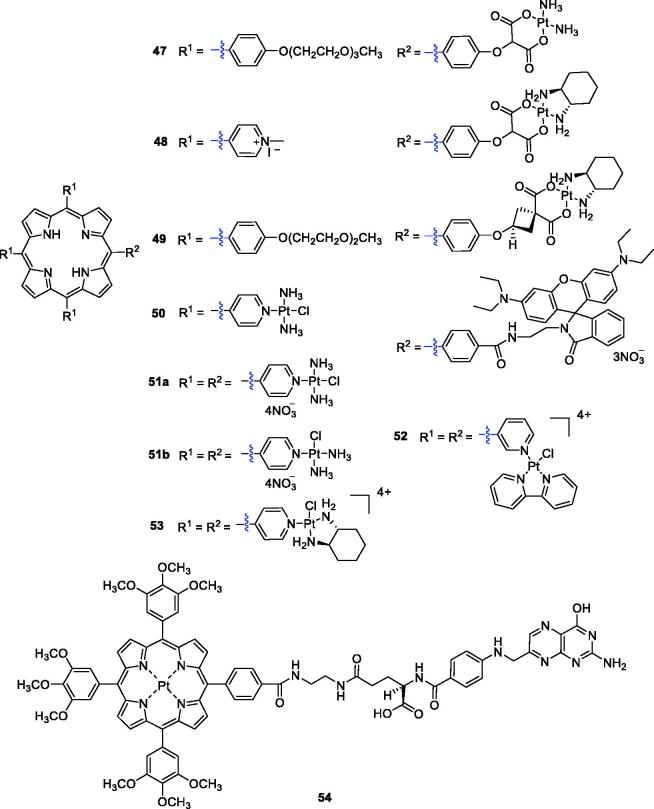
Porphyrin–platinum complexes **47–54**.

Brunner et al.^96^ synthesised porphyrin–platinum conjugates and made great progress in this field. To overcome the shortcomings of light penetration depth, Lottner et al.^97^ replaced hematoporphyrin with a tetraarylporphyrin and increased the penetration depth by redshifting the illumination wavelength. These compounds increased the anti-tumour activity of the platinum group through additional photoinduced toxicity. Among all the synthesised compounds, the most active compound was a tetraarylporphyrin–platinum conjugate with diamine and (*RR*/*SS*)-*trans*-1,2-diaminocyclohexane ligands (**47**). Study results revealed that the enhanced activity of compound **47** compared with the hematoporphyrin analogue was due to a redshift in the wavelength of the irradiation. Song et al.^98^ designed and synthesised a new series of DNA binding 5,10,15-tri(*N*-methyl-4-pyridiniumyl)porphyrin (TrisMPyP)-platinum(II) conjugates, in which different spacer ligands were used for appropriate coordination to platinum(II) complexes. Additional study confirmed that the anti-tumour activity of compound **48** (T/C% = 294) was superior to that of cisplatin (T/C%, 184) and approximately 1.6 times more potent than that of cisplatin against leukaemia L1210 cells (T/C values express the relative inhibitory activity of a compound on cell growth compared with the solvent reference). After etherification with diethyl cyclobutanedicarboxylate and subsequent ester hydrolysis, Brunner et al.^99^ prepared tetraarylporphyrin–platinum complexes combined with platinum fragments. All the compounds showed significant cytotoxic and phototoxic effects contributed from the platinum and porphyrin structures, and the antiproliferative activity of complex **49** even exceeded that of cisplatin (9.8-fold) (Figure 13).

Zhu et al.^17^ designed and synthesised a novel PDT agent (**50**) for cancer therapy (Figure 13) that could rapidly generate singlet oxygen with low dark cytotoxicity and exhibited a concentration-dependent photocytotoxicity against HeLa cells (IC_50_ = 3.38 µM under a yellow light dose of 4 J/cm^2^). From commercially available 5,10,15,20-tetra(4-pyridyl)porphyrin and platinum complexes with different substituents, Naik et al.^100^ reported the synthesis of tetraplatinated porphyrins with *in vitro* light-induced anticancer activity. They identified a very potent compound, porphyrin **51a**, which showed promising photocytotoxic properties and was extremely toxic against human cancerous cell lines upon irradiation with light at 420 nm, 6.95 J/cm^2^ (HeLa: IC_50_ = 40 nM; A2780: IC_50_ =21 nM; CP70: IC_50_ = 19 nM). Further evaluations suggested that tetraplatinated porphyrin complexes could be developed as PDT anticancer agents for drug-resistant malignancies.

In another study, Tasso et al.^101^ synthesised isomers of free-base meso-tetra(pyridyl)porphyrins derivatives with [PtCl(bipy)]^+^ moieties, and an array of assays was performed to evaluate its photophysics and anticancer potential using the HeLa cell line. The results confirmed that isomer **52** showed less efficient electronic communication between the Pt(II) moieties. Similarly, the total charge distribution of isomer **52** gave the molecule higher amphiphilicity, resulting in greater membrane affinity and greater cellular uptake. It was also reported that compound **52** (LD_50_ = 25 nM) was two times more photocytotoxic than the *meta* substituted porphyrin (LD_50_ = 50 nM) under the optimum dose of 1 J/cm^2^ (522 nm). More recently, Hu et al.^102^ carried out the synthesis of a novel tetracationic porphyrin–platinum (II) conjugate (**53**). This derivative displayed low dark cytotoxicity and excellent photocytotoxicity when irradiated with 6 J/cm^2^ at 570 nm (Colon26: IC_50_ = 0.17 µM; Sarcoma180: IC_50_ = 0.25 µM) and showed reasonable water solubility and high singlet oxygen quantum yield. In a therapy (PDT) assay, compound **53** completely killed tumour tissues *in vivo*, rather than simply inhibiting the tumour growth. No recurrence occurred 18 days after a single administration. The results for compound **53** provide a reference for clinical application of tumour PDT in the future.

Yang et al.^103^ designed and synthesised a platinum porphyrin–folate conjugate (**54**) as an efficient PS for tumour-targeting PDT (Figure 13)**.** Compound **54** showed significant therapeutic efficacy against HeLa cells in a dose-dependent manner, and the IC_50_ value was approximately 5.78 µM (irradiation with 4 J/cm^2^ of 500 nm light). Through FR-mediated endocytosis due to folate coupling, compound **54** could specifically target cancer cells overexpressing FR (HeLa cells).

**Figure 14.**
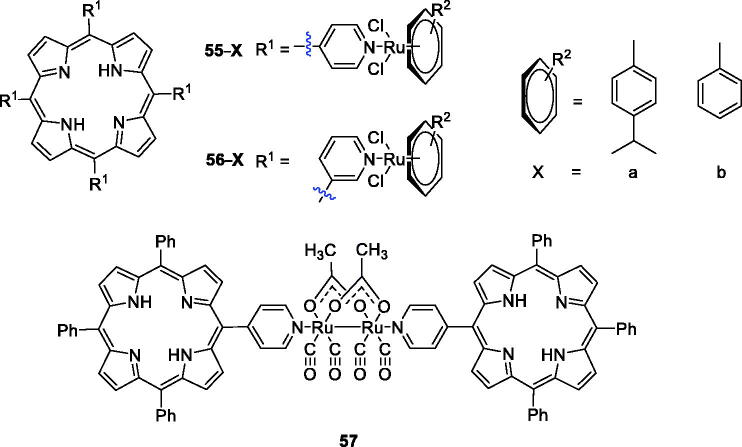
Porphyrin–ruthenium conjugates **55–57**.

Platinum derivatives are toxic to normal cells. Therefore, the use of metal ruthenium is very appealing to overcome the drawbacks and allow interaction with DNA and proteins^104–106^. Schmitt et al.^105^ were interested in combining the photodynamic action of porphyrins with the cytotoxicity of arene ruthenium complexes and have achieved some success. The synthesis and characterisation of compounds **55a**–**b** (Figure 14) were reported, and the results showed that ruthenium facilitated uptake and highly active photosensitising activity under red light (652 nm) at an irradiation dose of only 5 J/cm^2^ in a human Me300 melanoma cell model, because exposure to only 5 J/cm^2^ of light induced 60–80% phototoxicity in melanoma cells. In their follow-up study, they synthesised a series of new arene ruthenium porphyrin compounds containing either one or four arene ruthenium units to better explore their mechanisms of action in human melanoma cells. With similar spectroscopic properties, the 3-pyridyl PS was more photosensitising than the 4-pyridyl PS at an equivalent degree of substitution. In particular, compound **56**, which consisted of four arene ruthenium groups, exerted better activity. Compound **56** induced cell death at 5 µM when the light dose was less than 0.5 J/cm^2^ at 652 nm^104^. In their third study, a diruthenium tetracarbonyl structure was chosen as the organometallic agent, and a complex with a sawhorse-like geometry and two axial directions was synthesised as the axial ligand in the porphyrin derivative substituent (**57**). Compound **57** (2.5 µM) demonstrated no dark cytotoxicity but showed good phototoxicity against HeLa and A2780 cells exposed to laser light at 652 nm, displaying an LD_50_ between 1.5 and 6.5 J/cm^2^ in these two cell lines and more than 15 J/cm^2^ in other cell lines. Furthermore, these types of porphyrin compounds were specific only to cancer cell lines of the female reproductive system and did not damage normal cells^107^.

**Figure 15.**
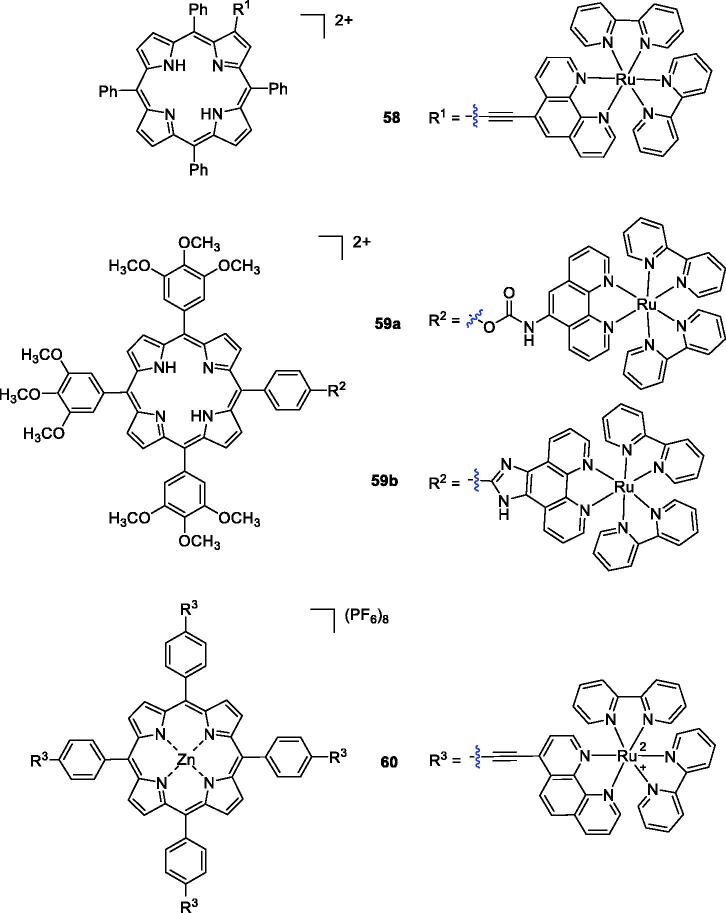
Porphyrin–ruthenium conjugates **58–60**.

In another study, Zhang et al.^108^ synthesised porphyrin derivatives containing Ru(II) polypyridyl-porphyrin and Zn(II) porphyrin structures and evaluated their cytotoxicity against human nasopharyngeal carcinoma HK-1 and cervical carcinoma HeLa cells. Among all the synthesised compounds, only compound **58** showed a high singlet oxygen quantum yield, rapid cellular uptake, low dark-cytotoxicity and potent photocytotoxicity (at 1 µM concentration and under a yellow light dose of 3 J/cm^2^, where 80% of the HK-1 cells incubated with Ru-L were killed; Figure 15). Then, a novel series of the Ru (polypyridyl)-based compounds was synthesised by Pan et al.^109^ using different linkers, and their photophysical properties were evaluated. Compounds **59a**–**b** exhibited high singlet oxygen quantum yields, and Ru(II) conjugate **59 b** (IC_50_ value of 9.6 µM at yellow light doses of 1 J/cm^2^) was the best PDT reagent against HeLa cells.

A novel porphyrin-core compound (**60**) was prepared by cross-coupling the terminal alkyne groups of meso-tetra(4-ethynylphenyl)porphyrin-Zn(II)(P-1) with a halogenated Ru(II)-phenanthroline complex using the Sonogashira reaction. Upon irradiation with 33 J/cm^2^ of 620–630 nm light, P-Ru killed 90% of SKBR-3 cells at a concentration of 1 µM. Notably, compound **60** induced a 77% decrease in cell viability at a concentration of only 0.25 µM^110^.

In recent years, due to the similarities between gold and platinum, several gold-based compounds have been synthesised and successfully evaluated as potential anti-cancer agents^111–113^. For example, novel gold (III) meso-tetraarylporphyrins complexes prepared by Che et al.^114^ showed significant cytotoxicities against human cancer cells, with IC_50_ values of 0.1 ∼ 1.5 µM. Among them, compound **61** showed the best effects (IC_50_ = 0.1 ∼ 0.8 µM), which were 100 times higher than that of cisplatin against human cancer cell lines, including multidrug- (KB-V1) and cisplatin-resistant cancer cells (CNE-1). In their subsequent work, they explored the cellular pharmacological properties of gold (III) porphyrin **61**. Cytotoxicity study of **61** demonstrated that the higher cytotoxicity of gold (III) porphyrin was not related to its photosensitising activity^115^.

Although this gold(III) tetraphenyl porphyrin showed high anticancer activity, it is not an approved clinical drug. To reduce its toxicity and improve activity, plenty of research work has been carried out to develop more efficient and selective derivatives of gold(III) tetraphenylporphyrin^116^. Chen et al.^116^ reported the introduction of organophosphorus into gold (III) tetraphenylporphyrins. The cytotoxic activities of these compounds were tested against SMMC-7721 human hepatic cancer cells and sarcoma 180 mouse cancer cells using the standard MTT method. 5-[4(Diisopropoxyphosphorylamino)]phenyl-10,15,20-triphenylporphyrinato gold(III) chloride (**62a**) showed more potent activity than cisplatin against sarcoma 180 mouse cancer cells (IC_50_ = 5.10 µM), while compound 5-[4(dipropoxyphosphorylamino)]phenyl 10,15,20-triphenylporphyrinato gold(III)chloride (**62b**) displayed the highest level of cytotoxic activity in SMMC-7721 human hepatic cancer cells (IC_50_ = 2.60 µM). Regretfully, none of these compounds exhibited more excellent anticancer activity than gold(III) tetraphenyl-porphyrin^117^^,^^118^.

Recently, Longevial et al.^119^ first used carbohydrates to anchor at the periphery of the porphyrin through the formation of metal–ligand bonds. Compound **63** could be a valuable agent for PDT applications because it contains three structural units with different functions: a free base porphyrin that functions as a PS; Au acting as connecting metal ion; and mannose serving as a targeting unit that also improves the compound water solubility. As demonstrated, the role of the mannose linked to Au^I^ was of crucial importance in improving the global photodynamic effect. Based on the combination principle drug design strategies, Hu et al.^120^ combined a porphyrin skeleton, Pt(II)-based chemotherapeutic drug with a metal ion gallium (III) to improve the hydrophilicity and increase tumour accumulation. Due to its heavy atom effect, large *p*Ka value, and localisation in the cytosol, the mixed-metal porphyrin Ga-4cisPtTPyP (**64)** exerted a high yield of singlet oxygen, more potent than the reference drug 4cisPtTPyP (**51 b**). In the *in vivo* PDT experiments, compound **64** almost completely inhibited tumour growth in a short time, especially in colon cancer 26 and sarcoma 180 cells, and the IC_50_ values were 0.12 and 0.08 µΜ, respectively, upon illumination with a 50 W LED light. Based on this evidence, compound **64** may be a very promising anticancer candidate for PDT.

**Figure 16.**
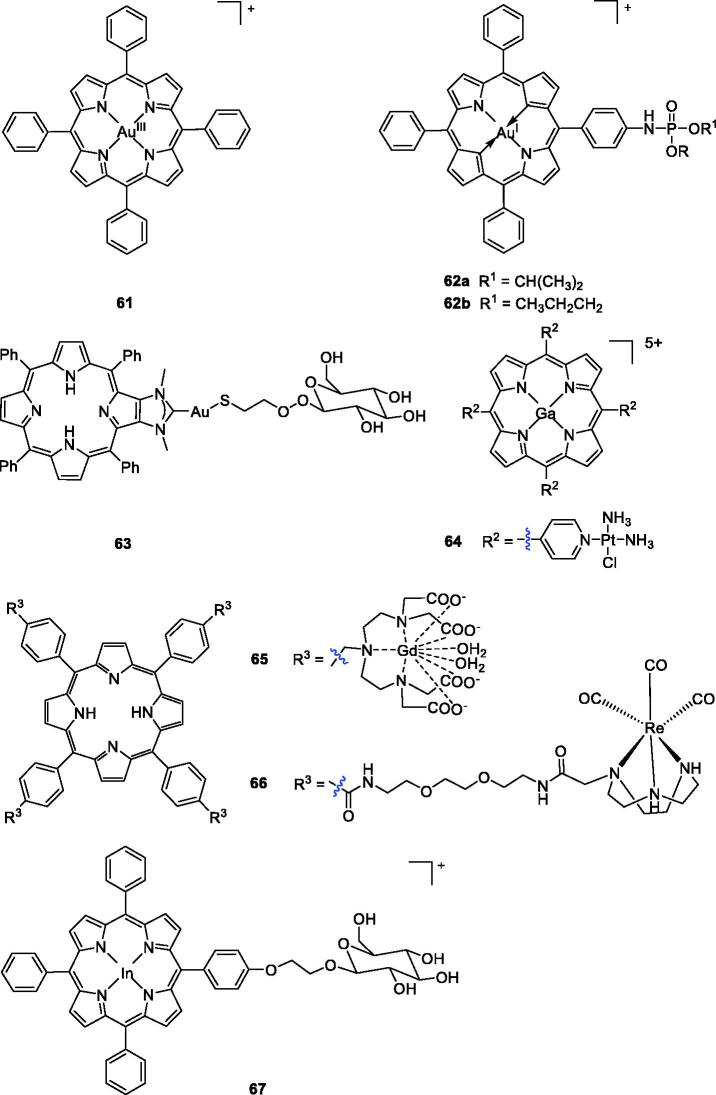
Porphyrin–gold, gadolinium, indium, and rhenium complexes **61–67**.

A novel series of anionic [Gd(DTTA)]-complexes with a porphyrin core were designed by Sour et al.^121^ The most potent compound (**65**) displayed appropriate photophysical properties and remarkable fluorescence quantum. Moreover, compound **65** was found to induce HeLa cell apoptosis (LD_50_ = 6 µM at 21 J/cm^2^, 636 nm). Indium porphyrins are also clinical PET agents, and the effective photodynamic activity of a few indium porphyrins has been reported^122^^,^^123^. Aiming to develop new bifunctional photosensitisers, they also described the synthesis of glucopyranose-conjugated indium porphyrins, their application in diagnosis and their PDT properties. Compound **67** exhibited the most potent photocytotoxicity (IC_50_ = 0.012 µM at 16 J/cm^2^, 663 nm) against COLO 679 cells^124^. In addition, Mion et al.^125^ described the synthesis of a series of Re-porphyrin conjugates, and compound **66** showed remarkable phototoxicity against HeLa cells, similar to compound **1**. It was noted that the rhenium fragment did not enhance phototoxicity, suggesting that other features (hydrophilicity, permanent positive charges, one flexible arm, etc.) played a major role in determining the phototoxic properties of the compounds. Notably, certain organometallic rhenium complexes have been found to possess interesting luminescence properties, and thus, their intracellular distribution could be observed by emission microscopy, which was helpful in understanding their mechanism of action (Figure 16).

### 2.4. Peptide-coupled porphyrins

The advantages of peptide-coupled PSs are as follows: (a) peptides are easily synthesised and structurally modified; (b) short synthetic peptides are ideal candidates for drug delivery due to their effective tissue penetration, selective binding and internalisation capacity; (c) peptides have virtually no cytotoxicity; and (d) peptides facilitate rapid access to the tumour site^126^.

Linear and cyclic-RGD peptides were conjugated to different types of PSs to promote selectivity and PDT in tumour cells^127^^,^^128^. The PpIX:cRGDfK conjugate was found to be a good PS in the integrin-positive human SiHa cell line *in vitro* and in a mouse CaNT tumour model *in vivo*^129^. In this study, investigators prepared a library based on RGD-porphyrin derivatives bearing a spacer arm and sugar units. Among all the compounds in the library, compound **68a** showed lower biological activity, which might be due to its poor solubility, while the photoactivity of porphyrin **68b** was similar to that of compound **1**^130^.

Sibrian-Vazquez et al.^131^ demonstrated that the number, nature, sequence of amino acids, and presence of a chelated metal ion affect the cellular uptake of the conjugates. First, they reported 4-aminomeso-tetraphenylporphyrin derivatives bearing charged amino-acids and peptide moieties, such as lysine and arginine residues. All the compounds had low dark cytotoxicity (IC_50_ > 250 µM), and compound **69** with three consecutive arginine residues (R^2^ = RRRNH_2_ and KRRRNH_2_) exhibited much higher activity.

**Figure 17.**
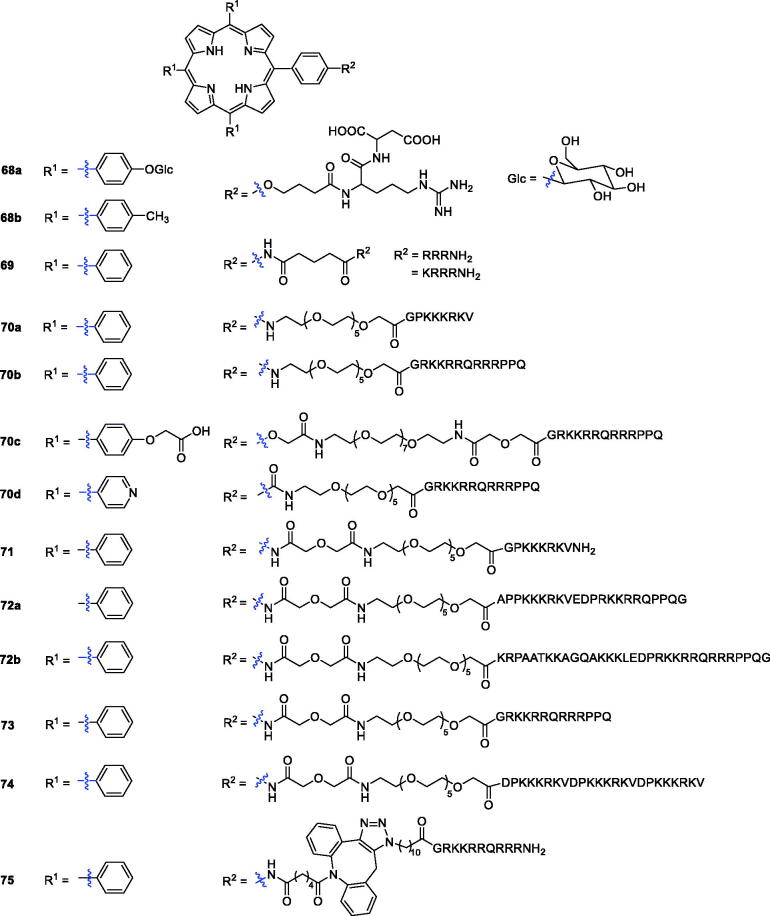
Peptide-coupled porphyrin derivatives **68–75**.

Combining porphyrin PSs with a carrier protein or peptide containing a nuclear localisation sequence (NLS) is an effective strategy. Therefore, the minimum NLS PKKKRKV from the large T antigen of the simian virus (SV40) has been studied^132^. In particular, the human immunodeficiency virus 1 transcription activator (HIV-1 Tat) has been shown to promote cellular uptake of many drugs and macromolecules^133^. Following the above principle, porphyrin–peptide derivatives bearing the SV40 NLS or the fusogenic HIV-1 Tat 48–60 peptide sequences were prepared using low molecular weight PEG molecules as linkers. At the same time, a hydrophilic group (carboxyl or pyridyl) was introduced into the periphery of the porphyrin macrocycle. Porphyrin–peptide conjugates **70b**–**d,** bearing the HIV-1 Tat 48–60 peptide, were more efficiently delivered into the cells than those containing the SV40 peptide. However, the hydrophobic conjugates **70a**–**b** were found to be highly phototoxic (IC_50_ = 1.5 and 2.3 µM, respectively) against HEp2 cells using a 100 W halogen lamp with a total light dose of approximately 1 J/cm^2^ (Figure 17)^134^.

Sibrian-Vazquez et al.^135^ reported the synthesis and evaluation of porphyrin–peptide compounds conjugated with the SV40 nuclear localisation sequence or a fusogenic peptide (HIV-1Tat 40–60 or octa-arginine) linked to low molecular weight poly(ethylene glycol), and their activity as PDT agents was examined against human HEp2 cells. With the help of *in vitro* studies, it was clearly observed that compound **71** displayed an IC_50_ value of 1.5 µM in HEp2 cells after exposure to light from a 100 W halogen lamp at 0.5 J/cm^2^ (Figure 17). Sibrian-Vazquez et al.^136^ also presented the synthesis of some novel porphyrin–peptide conjugates with one linear bifunctional sequence having a cell penetrating peptide (CPP) and NLS. It was found that the accumulation of all conjugates in human HEp2 cells was much greater than that of their porphyrin–PEG precursor. The conjugates **72a**–**b** (Figure 17) bearing a NLS-CPP accumulated most in cells and were the most phototoxic (IC_50_ = 7 µM exposed to light from a 100 W halogen lamp at 1 J/cm^2^).

Porphyrin derivatives with the SV40 NLS have been reported and have shown increased photosensitising activity in comparison with the corresponding unconjugated porphyrins^135^. Although *in vitro* studies have shown that porphyrin–peptide PSs containing CPP (HIV-1 or penetration) can significantly increase cell absorption and phototoxicity compared with unconjugated porphyrins, they are enzymatically hydrolysed and significantly lack tumour specificity^136^^,^^137^. Sehgal et al.^138^ studied the impact of the peptide sequences in the porphyrin ring. Compounds containing CPP, NLS, or bifunctional CPP-NLS or NLS-CPP sequences exhibited photodynamic activity against PC-3M human prostate cancer cells. The porphyrin-HIV-1 Tat (48–60) **73** displayed more potent phototoxicity (IC_50_ = 0.40 µM, exposed to light from a 100 W halogen lamp at 1 J/cm^2^) than compound **2**. The most active porphyrin-HIV-1 Tat (48–60) **73** was further evaluated in an *in vivo* biodistribution investigation using SCID mice bearing PC-3M tumours. It was confirmed to be more selectively localised in tumours than the hematoporphyrin derivative.

Previous studies have found that porphyrin peptide conjugates with multiple NLSs can increase the affinity for tumour cells, thereby increasing photodynamic activity. Therefore, Sibrian-Vazquez et al.^139^ reported the synthesis of porphyrin–NLS conjugates, among which the smallest sequence was PKKKRKV connected to PEG or 5-carbon spacers, and evaluated their activity against human carcinoma HEp2 cells. The results showed that compound **74** was the most phototoxic, while the tetra-NLS conjugates symmetrically substituted around the porphyrin ring were non-phototoxic and accumulated the least in cells.

Dondi et al.^140^ reported the synthesis of a series of novel peptide-porphyrin conjugates using a hydrophobic porphyrin and polycationic hydrophilic peptide components connected through a triazole-based linker and evaluated their activities against MCF-7 cells and MC28 cells. Compound **75**, with a triazole-based linker, was found to be well-suited for light-triggered drug delivery and was the most promising candidate as a lead compound for an anticancer drug (LD_50_ = 37 ± 2 nM and illuminated with a blue lamp with peak emission at 420 nm and 7 Mw/cm^2^ output for 7 min).

### 2.5. Nanotechnology and nanochemistry

In the past few decades, the rapid development of nanotechnology has brought new opportunities to improve the application of porphyrin-based PDT *in vivo*. There are three main reasons why nanotechnology is attractive in PDT: (1) targeting potential increases the concentration of the PS at the target site, which increases the accumulation of porphyrins in tumours, and reduces damage to normal tissues/cells; (2) nanoparticles (NPs) can improve the water solubility and light stability of hydrophobic PSs; and (3) NPs can maintain a constant rate of PS delivery at the desired sites due to zero-order release kinetics^141^. Nanoparticle-mediated porphyrin delivery strategies include encapsulation (metal-organic frameworks^142^, polymeric micelles^143^, mesoporous silica nanoshells^144^) covalent conjugation^145^, and self-assembly^146^. For example, Bretin et al.^147^ demonstrated the strong anticancer efficacy and tumour-targeting capability of 5–(4-hydroxyphenyl)-10,15,20-triphenylporphyrin (TPPOH) *in vitro* and *in vivo* and improved the non-toxic anti-cancer effect of xylan-TPPOH conjugate (TPPOH-X) SNPs *in vivo* by improving tumour targeting. Pan et al.^148^ demonstrated the functionalisation of two new zinc metalised porphyrins with two symmetrical phenylethyl groups, showing strong absorbance at 677 nm and 694 nm. This result indicated that the self-assembled porphyrins can be taken up by cancer cells, leading to low dark toxicity, high phototoxicity and strong cell fluorescence. Despite tremendous efforts to develop modified nanosystems for effective PDT for cancer treatment, searching for a nanostructured drug delivery system based on surface-functionalised NPs that combine targeted molecular recognition of tumours with reactive singlet oxygen production by PSs under PDT irradiation remains a challenge. Such system would be considered to be biosafe in clinical settings and thus requires further investigation^141^.

## 3. Conclusions and perspectives

During the past several years, most research has focussed on improving the photophysical properties of old-style PSs and/or improving their tumour targeting capability through different structural modifications, such as combination with other molecules, metallisation and nanotechnology applications.

Some examples show PSs that are highly active in tumour cell models (in theory) and maybe not affect tumours in animal models (in the experiment). Frimayanti et al.^149^ reported the use of the quantitative structure–activity relationship (QSAR) method to develop a model that could correlate the structural features of cyclic tetrapyrrole-based compounds with their photodynamic therapy (PDT) activity, but some compounds that were flagged as theoretically active PSs by this model did not show good PDT activities under experimental conditions. PDT is a complex treatment that combines drugs with light. It depends on the chemical and phytochemical profiles of the PS, the dose of the PS, the wavelength of irradiation light and the oxidation state of the tissue. Therefore, in addition to developing new PSs, it is also very important to establish an appropriate PDT treatment plan. At present, the effects of PSs after structural modification cannot be directly compared because the assays are performed under different parameters. A standard needs to be established to select better PSs. The solution to this intractable problem is to conduct reasonable mechanistic studies and find a way to predict the efficacy of a PS based on its photophysical measurements^11^.

In addition, the earliest reported photoactive drugs were natural products. Therefore, further research can also focus on natural PSs (furocoumarin, polyacetylene molecules and thiophenes, curcumin, alkaloids, and anthraquinones). Moreover, combining natural photoactive substances with synthetic PSs may lead to a major breakthrough in PDT^150^.

## References

[1] Wong MCS, Lao XQ, Ho KF, et al. Incidence and mortality of lung cancer: global trends and association with socioeconomic status. Sci Rep 2017;7:14300. 29085026 10.1038/s41598-017-14513-7 PMC5662733

[2] Siegel RL, Miller KD, Jemal A. Cancer statistics, 2020. CA Cancer J Clin 2020;70:7–30. 31912902 10.3322/caac.21590

[3] Yano S, Hirohara S, Obata M, et al. Current states and future views in photodynamic therapy. J Photochem Photobiol C 2011;12:46–67.

[4] Dougherty TJ, Kaufman JE, Goldfarb A, et al. Photoradiation therapy for the treatment of malignant tumors. Cancer Res 1978;38:2628–35. 667856

[5] Habermeyer B, Guilard R. Some activities of PorphyChem illustrated by the applications of porphyrinoids in PDT, PIT and PDI. Photochem Photobiol Sci 2018;17:1675–90. 30109347 10.1039/c8pp00222c

[6] Dolmans DE, Fukumura D, Jain RK. Photodynamic therapy for cancer. Nat Rev Cancer 2003;3:380–7. 12724736 10.1038/nrc1071

[7] Li X, Lee S, Yoon J. Supramolecular photosensitizers rejuvenate photodynamic therapy. Chem Soc Rev 2018;47:1174–88. 29334090 10.1039/c7cs00594f

[8] Levy J, Photofrin-PDT from bench to bedside: some lessons learned. In: Pandey RK, Dougherty TJ, Kessel D, eds. Handbook of photodynamic therapy: updates on recent applications of porphyrin-based compounds. Singapore: World Scientific Publishing; 2016.

[9] Agostinis P, Berg K, Cengel KA, et al. Photodynamic therapy of cancer: an update. CA Cancer J Clin 2011;61:250–81. 21617154 10.3322/caac.20114 PMC3209659

[10] Gomes A, Neves M, Cavaleiro J. Cancer, photodynamic therapy and porphyrin-type derivatives. An Acad Bras Cienc 2018;90:993–1026. 29873666 10.1590/0001-3765201820170811

[11] Castano AP, Demidova TN, Hamblin MR. Mechanisms in photodynamic therapy: part one-photosensitizers, photochemistry and cellular localization. Photodiagn Photodyn Ther 2004;1:279–93. 10.1016/S1572-1000(05)00007-4 PMC4108220 25048432

[12] Ethirajan M, Chen Y, Joshi P, Pandey RK. The role of porphyrin chemistry in tumor imaging and photodynamic therapy. Chem Soc Rev 2011;40:340–62. 20694259 10.1039/b915149b

[13] Martinez D, Mroz P, Thunshelle C, Hamblin MR. Design features for optimization of tetrapyrrole macrocycles as antimicrobial and anticancer photosensitizers. Chem Biol Drug Des 2017;89:192–206. 28205400 10.1111/cbdd.12792 PMC5319686

[14] Xiong Y, Tian XD, Ai HW. Molecular tools to generate reactive oxygen species in biological systems. Bioconjugate Chem 2019;30:1297–303. 10.1021/acs.bioconjchem.9b00191 PMC6528174 30986044

[15] Liu C, Dobhal MP, Ethirajan M, et al. Highly selective synthesis of the ring-B reduced chlorins by ferric chloride-mediated oxidation of bacteriochlorins: effects of the fused imide vs isocyclic ring on photophysical and electrochemical properties. J Am Chem Soc 2008;130:14311–23. 18828591 10.1021/ja8050298

[16] Feng X, Shi Y, Xie L, et al. Synthesis, characterization, and biological evaluation of a porphyrin-based photosensitizer and its isomer for effective photodynamic therapy against breast cancer. J Med Chem 2018;61:7189–720. 30048595 10.1021/acs.jmedchem.8b00547

[17] Zhu S, Yao S, Wu F, et al. Platinated porphyrin as a new organelle and nucleus dual-targeted photosensitizer for photodynamic therapy. Org Biomol Chem 2017;15:5764–71. 28660264 10.1039/c7ob01003f

[18] Stacey OJ, Pope SJA. New avenues in the design and potential application of metal complexes for photodynamic therapy. RSC Adv 2013;3:25550–64.

[19] Zhang J, Jiang CS, Longo JPF, et al. An updated overview on the development of new photosensitizers for anticancer photodynamic therapy. Acta Pharm Sin B 2018;8:137–46. 29719775 10.1016/j.apsb.2017.09.003 PMC5925394

[20] Bůžek D, Zelenka J, Ulbrich P, et al. Nanoscaled porphyrinic metal–organic frameworks: photosensitizer delivery systems for photodynamic therapy. J Mater Chem B 2017;5:1815–21. 32263922 10.1039/c6tb03230c

[21] Hynek J, Ondrušová S, Bůžek D, et al. Postsynthetic modification of a zirconium metal-organic framework at the inorganic secondary building unit with diphenylphosphinic acid for increased photosensitizing properties and stability. Chem Commun 2017;53:8557–60. 10.1039/c7cc05068b 28714507

[22] Boni LD, Monteiro CJP, Mendonça CR, et al. Influence of halogen atoms and protonation on the photophysical properties of sulfonated porphyrins. Chem Phys Lett 2015;633:146–51.

[23] Kessel D, Thompson P, Saatio K, Nantwi KD. Tumor localization and photosensitization by sulfonated derivatives of tetraphenylporphine. Photochem Photobiol 1987;45:787–90. 3628502 10.1111/j.1751-1097.1987.tb07883.x

[24] Winkelman JW, Collins GH. Neurotoxicity of tetraphenylporphinesulfonate TPPS4 and its relation to photodynamic therapy. Photochem Photobiol 1987;46:801–7. 3441503 10.1111/j.1751-1097.1987.tb04851.x

[25] Thomas AP, Saneesh Babu PS, Ramakrishnan S, et al. meso-Tetrakis(p-sulfonatophenyl)N-confused porphyrin tetrasodium salt: a potential sensitizer for photodynamic therapy. J Med Chem 2012;55:5110–20. 22582931 10.1021/jm300009q

[26] Hynek J, Koncosova M, Zelenka J, et al. Phosphinatophenylporphyrins tailored for high photodynamic efficacy. Org Biomol Chem 2018;16:7274–81. 30259016 10.1039/c8ob01984c

[27] You Y, Gibson SL, Hilf R, et al. Water soluble, core-modified porphyrins. 3. Synthesis, photophysical properties, and in vitro studies of photosensitization, uptake, and localization with carboxylic acid-substituted derivatives. J Med Chem 2003;46:3734–47. 12904078 10.1021/jm030136i

[28] Stilts CE, Nelen MI, Hilmey DG, et al. Water-soluble, core-modified porphyrins as novel, longer-wavelength-absorbing sensitizers for photodynamic therapy. J Med Chem 2000;43:2403–10. 10882367 10.1021/jm000044i

[29] Wilkinson F, Helman WP, Ross AB. Quantum yields for the photosensitized formation of the lowest electronically excited singlet state of molecular oxygen in solution. J Phys Chem Ref Data 1993;22:113–262.

[30] Hilmey DG, Abe M, Nelen M, et al. Water-soluble, core-modified porphyrins as novel, longer-wavelength-absorbing sensitizers for photodynamic therapy. II. Effects of core heteroatoms and meso-substituents on biological activity. J Med Chem 2002;45:449–61. 11784149 10.1021/jm0103662

[31] McMillin DR, Shelton AH, Bejune SA, et al. Understanding binding interactions of cationic porphyrins with B-form DNA. Coord Chem Rev 2005;249:1451–9.

[32] Slomp AM, Barreira SMW, Carrenho LZB, et al. Photodynamic effect of meso-(aryl)porphyrins and meso-(1-methyl-4-pyridinium)porphyrins on HaCaT keratinocytes. Bioorg Med Chem Lett 2017;27:156–61. 27956348 10.1016/j.bmcl.2016.11.094

[33] Jensen TJ, Vicente MGH, Luguya R, et al. Effect of overall charge and charge distribution on cellular uptake, distribution and phototoxicity of cationic porphyrins in HEp2 cells. J Photochem Photobiol B 2010;100:100–11. 20558079 10.1016/j.jphotobiol.2010.05.007 PMC3161426

[34] Kumar D, Shekar KPC, Mishra B, et al. Cationic porphyrin-quinoxaline conjugate as a photochemically triggered novel cytotoxic agent. Bioorg Med Chem Lett 2013;23:3221–4. 23639546 10.1016/j.bmcl.2013.03.126

[35] Jelovica M, Grbcic P, Muskovic M, et al. In vitro photodynamic activity of N-methylated and N-oxidised tripyridyl porphyrins with long alkyl chains and their inhibitory activity in sphingolipid metabolism. ChemMedChem 2018;13:360–72. 29381258 10.1002/cmdc.201700748

[36] Harris PA, Cheung M, Hunter IIR, et al. Discovery and evaluation of 2-anilino-5-aryloxazoles as a novel class of VEGFR2 kinase inhibitors. J Med Chem 2005;48:1610–9. 15743202 10.1021/jm049538w

[37] Zheng YM, Wang K, Li T, et al. Synthesis, singlet oxygen photogeneration and DNA photocleavage of porphyrins with nitrogen heterocycle tails. Molecules 2011;16:3488–98. 21522082 10.3390/molecules16053488 PMC6263290

[38] Sari MA, Battioni JP, Dupre D, et al. Interaction of cationic porphyrins with DNA: importance of the number and position of the charges and minimum structural requirements for intercalation. Biochemistry 1990;29:4205–15. 2361139 10.1021/bi00469a025

[39] Dutikova YV, Borisova OF, Shchyolkina AK, et al. 5,10,15,20-Tetra-(N-methyl-3-pyridyl)porphyrin destabilizes the antiparallel telomeric quadruplex d(TTAGGG)_4_. Mol Biol 2010;44:823–31. 21090248

[40] Antoni PM, Naik A, Albert I, et al. (Metallo)porphyrins as potent phototoxic anti-cancer agents after irradiation with red light. Chem Eur J 2015;21:1179–83. 25421757 10.1002/chem.201405470

[41] Yoho J, Wogensthal K, Bennett TL, et al. Water-soluble zinc porphyrin capable of light-induced photocleavage of DNA: cell localization studies in Drosophila melanogaster and light activated treatment of lung cancer cells. Eur J Inorg Chem 2017;2017:153–9.

[42] Rice KP, Penketh PG, Shyam K, Sartorelli AC. Differential inhibition of cellular glutathione reductase activity by isocyanates generated from the antitumor prodrugs Cloretazine™ and BCNU. Biochem Pharmacol 2005;69:1463–72. 15857610 10.1016/j.bcp.2005.02.016

[43] Elms J, Beckett PN, Griffin P, Curran AD. Mechanisms of isocyanate sensitisation. An in vitro approach. Toxicol in Vitro 2001;15:631–4. 11698162 10.1016/s0887-2333(01)00078-9

[44] Silva P, Fonseca SM, Arranja CT, et al. A new nonconjugated naphthalene derivative of meso-tetra-(3-hydroxy)-phenyl-porphyrin as a potential sensitizer for photodynamic therapy. Photochem Photobiol 2010;86:1147–53. 20553404 10.1111/j.1751-1097.2010.00764.x

[45] William B, Reinhold T. Silicon chemistry as a novel source of chemical diversity in drug design. Curr Opin Drug Discov Devel 2003;6:526–43. 12951816

[46] Jiang XJ, Lo PC, Yeung SL, et al. A pH-responsive fluorescence probe and photosensitiser based on a tetraamino silicon (IV) phthalocyanine. ChemCommun 2010;46:3188–90. 10.1039/c000605j 20424769

[47] Horiuchi H, Hosaka M, Mashio H, et al. Silylation improves the photodynamic activity of tetraphenylporphyrin derivatives in vitro and in vivo. Chem Eur J 2014;20:6054–60. 24710805 10.1002/chem.201303120

[48] Morlière P, Momenteau M, Candide C, et al. Synthesis, cellular uptake of, and cell photo-sensitization by a porphyrin bearing a quinoline group. J Photochem Photobiol B 1990;5:49–67. 2111393 10.1016/1011-1344(90)85005-h

[49] Costa LD, Silva JA, Fonseca SM, et al. Photophysical characterization and in vitro phototoxicity evaluation of 5,10,15,20-tetra(quinolin-2-yl)porphyrin as a potential sensitizer for photodynamic therapy. Molecules 2016;21:439. 27043519 10.3390/molecules21040439 PMC6273532

[50] Rangasamy S, Ju H, Um S, et al. Mitochondria and DNA targeting of 5,10,15,20-tetrakis(7-sulfonatobenzo[b]thiophene) porphyrin-induced photodynamic therapy via intrinsic and extrinsic apoptotic cell death. J Med Chem 2015;58:6864–74. 26295496 10.1021/acs.jmedchem.5b01095

[51] Frederiksen LJ, Sullivan R, Maxwell LR, et al. Chemosensitization of cancer in vitro and in vivo by nitric oxide signaling. Clin Cancer Res 2007;13:2199–206. 17404104 10.1158/1078-0432.CCR-06-1807

[52] Liu WK, Liu CZ, Gong CJ, et al. Porphyrins containing nitric oxide donors: synthesis and cancer cell-oriented NO release. Bioorg Med Chem Lett 2009;19:1647–9. 19233650 10.1016/j.bmcl.2009.02.005

[53] Li JW, Wu ZM, Magetic D, et al. Antitumor effects evaluation of a novel porphyrin derivative in photodynamic therapy. Tumour Biol 2015;36:9685–92. 26152290 10.1007/s13277-015-3745-z

[54] Stefano B, Enrico C, Stefania C, et al. Photodynamic effects of porphyrin and chlorin photosensitizers in human colon adenocarcinoma cells. Bioorg Med Chem 2004;12:4853–60. 15336264 10.1016/j.bmc.2004.07.011

[55] Hudson R, Savoie H, Boyle RW. Lipophilic cationic porphyrins as photodynamic sensitisers – synthesis and structure-activity relationships. Photodiagn Photodyn Ther 2005;2:193–6. 10.1016/S1572-1000(05)00065-7 25048769

[56] Liao PY, Wang XR, Gao YH, et al. Synthesis, photophysical properties and biological evaluation of β-alkylaminoporphyrin for photodynamic therapy. Bioorg Med Chem 2016;24:6040–7. 27713013 10.1016/j.bmc.2016.09.060

[57] Wang K, Poon CT, Choi CY, et al. Synthesis, circular dichroism, DNA cleavage and singlet oxygen photogeneration of 4-amidinophenyl porphyrins. J Porphyrins Phthalocyanines 2012;16:85–92.

[58] Zhu S, Wu F, Wang K, et al. Photocytotoxicity, cellular uptake and subcellular localization of amidinophenylporphyrins as potential photodynamic therapeutic agents: An in vitro cell study. Bioorg Med Chem Lett 2015;25:4513–7. 26338364 10.1016/j.bmcl.2015.08.072

[59] Greenwald RB. PEG drugs: an overview. J Control Release 2001;74:159–71. 11489492 10.1016/s0168-3659(01)00331-5

[60] Sibrian-Vazquez M, Jensen TJ, Vicente M. Synthesis and cellular studies of PEG-functionalized meso-tetraphenylporphyrins. J Photochem Photobiol B 2007;86:9–21. 16987669 10.1016/j.jphotobiol.2006.08.004

[61] Králová J, Bříza T, Moserová I, et al. Glycol porphyrin derivatives as potent photodynamic inducers of apoptosis in tumor cells. J Med Chem 2008;51:5964–73. 18788727 10.1021/jm8002119

[62] Schneider R, Schmitt F, Frochot C, et al. Design, synthesis, and biological evaluation of folic acid targeted tetraphenylporphyrin as novel photosensitizers for selective photodynamic therapy. Bioorg Med Chem 2005;13:2799–808. 15781391 10.1016/j.bmc.2005.02.025

[63] Stallivieri A, Colombeau L, Jetpisbayeva G, et al. Folic acid conjugates with photosensitizers for cancer targeting in photodynamic therapy: synthesis and photophysical properties. Bioorg Med Chem 2017;25:1–10. 27769669 10.1016/j.bmc.2016.10.004

[64] Lang K, Král VR, Kapusta P, et al. Photoinduced electron transfer within porphyrin–cyclodextrin conjugates. Tetrahedron Lett 2002;43:4919–22.

[65] Vyas A, Saraf S, Saraf S. Cyclodextrin based novel drug delivery systems. J Incl Phenom Macrocycl Chem 2008;62:23–42.

[66] Zhang J, Ma PX. Cyclodextrin-based supramolecular systems for drug delivery: recent progress and future perspective. Adv Drug Deliv Rev 2013;65:1215–33. 23673149 10.1016/j.addr.2013.05.001 PMC3885994

[67] Carofiglio T, Fornasier R, Lucchini V, et al. Synthesis, characterization, and supramolecular properties of a hydrophilic porphyrin–beta-cyclodextrin conjugate. J Org Chem 2000;65:9013–21. 11149845 10.1021/jo0010678

[68] Kralova J, Synytsya A, Pouckova P, et al. Novel porphyrin conjugates with a potent photodynamic antitumor effect: differential efficacy of mono- and bis-β-cyclodextrin derivatives in vitro and in vivo. Photochem Photobiol 2006;82:432–8. 16613522 10.1562/2005-05-06-RA-516

[69] Thomas T, Thomas TJ. Polyamine metabolism and cancer. J Cell Mol Med 2003;7:113–26. 12927050 10.1111/j.1582-4934.2003.tb00210.x PMC6740079

[70] Sol V, Lamarche F, Enache M, et al. Polyamine conjugates of meso-tritolylporphyrin and protoporphyrin IX: potential agents for photodynamic therapy of cancers. Bioorg Med Chem 2006;14:1364–77. 16263292 10.1016/j.bmc.2005.09.071

[71] Fidanzi-Dugas C, Liagre B, Chemin G, et al. Analysis of the in vitro and in vivo effects of photodynamic therapy on prostate cancer by using new photosensitizers, protoporphyrin IX-polyamine derivatives. Biochim Biophys Acta 2017;1861:1676–90. 10.1016/j.bbagen.2017.02.003 28188858

[72] Sarrazy V, Garcia G, Mbakidi JP, et al. Photodynamic effects of porphyrin–polyamine conjugates in human breast cancer and keratinocyte cell lines. J Photochem Photobiol B 2011;103:201–6. 21478031 10.1016/j.jphotobiol.2011.03.005

[73] Lupu M, Maillard P, Mispelter J, et al. A glycoporphyrin story: from chemistry to PDT treatment of cancer mouse models. Photochem Photobiol Sci 2018;17:1599–611. 29855017 10.1039/c8pp00123e

[74] Laville I, Figueiredo T, Loock B, et al. Synthesis, cellular internalization and photodynamic activity of glucoconjugated derivatives of tri and tetra(meta-hydroxyphenyl)chlorins. Bioorg Med Chem 2003;11:1643–52. 12659750 10.1016/s0968-0896(03)00050-6

[75] Desroches MC, Bautista-Sanchez A, Lamotte C, et al. Pharmacokinetics of a tri-glucoconjugated 5,10,15-(meta)-trihydroxyphenyl-20-phenyl porphyrin photosensitizer for PDT. A single dose study in the rat. J Photochem Photobiol B 2006;85:56–64. 16765603 10.1016/j.jphotobiol.2006.03.008

[76] Laville I, Pigaglio S, Blais JC, et al. Photodynamic efficiency of diethylene glycol-linked glycoconjugated porphyrins in human retinoblastoma cells. J Med Chem 2006;49:2558–67. 16610799 10.1021/jm0580151

[77] Maillard P, Loock B, Grierson DS, et al. In vitro phototoxicity of glycoconjugated porphyrins and chlorins in colorectal adenocarcinoma (HT29) and retinoblastoma (Y79) cell lines. Photodiagn Photodyn Ther 2007;4:261–8. 10.1016/j.pdpdt.2007.05.001 25047563

[78] Ashry E, Awad LF, Atta AI. Synthesis and role of glycosylthio heterocycles in carbohydrate chemistry. Tetrahedron 2006;62:2943–98.

[79] Sylvain I, Zerrouki R, Granet R, et al. Synthesis and biological evaluation of thioglycosylated porphyrins for an application in photodynamic therapy. Bioorg Med Chem 2002;10:57–69. 11738607 10.1016/s0968-0896(01)00255-3

[80] Kaldapa C, Blais JC, Carré V, et al. Synthesis of new glycosylated neutral and cationic porphyrins dimers. Tetrahedron Lett 2000;41:331–5.

[81] Ahmed S, Davoust E, Savoie H, et al. Thioglycosylated cationic porphyrins – convenient synthesis and photodynamic activity in vitro. Tetrahedron Lett 2004;45:6045–7.

[82] Stasio BD, Frochot C, Dumas D, et al. The 2-aminoglucosamide motif improves cellular uptake and photodynamic activity of tetraphenylporphyrin. Eur J Med Chem 2005;40:1111–22. 15963605 10.1016/j.ejmech.2005.04.007

[83] Ballut S, Makky A, Chauvin B, et al. Tumor targeting in photodynamic therapy. From glycoconjugated photosensitizers to glycodendrimeric one. Concept, design and properties. Org Biomol Chem 2012;10:4485–95. 22569817 10.1039/c2ob25181g

[84] Ballardini R, Colonna B, Gandolfi MT, et al. Porphyrin-containing glycodendrimers. Eur J Org Chem 2003;2003:288–94.

[85] Ballut S, Makky A, Loock B, et al. New strategy for targeting of photosensitizers. Synthesis of glycodendrimeric phenylporphyrins, incorporation into a liposome membrane and interaction with a specific lectin. ChemCommun 2009;8:224–6. 10.1039/b816128c 19099076

[86] Griegel S, Rajewsky MF, Ciesiolka T, Gabius HJ. Endogenous sugar receptor (lectin) profiles of human retinoblastoma and retinoblast cell lines analyzed by cytological markers, affinity chromatography and neoglycoprotein-targeted photolysis. Anticancer Res 1989;9:723–30. 2764517

[87] Wang ZJ, Chauvin B, Maillard P, et al. Glycodendrimeric phenylporphyrins as new candidates for retinoblastoma PDT: blood carriers and photodynamic activity in cells. J Photochem Photobiol B 2012;115:16–24. 22796430 10.1016/j.jphotobiol.2012.06.005

[88] Daghildjian K, Kasselouri A, N’Diaye M, et al. Mannose distribution in glycoconjugated tetraphenylporphyrins governs their uptake mechanism and phototoxicity. J Porphyrins Phthalocyanines 2019;23:175–84.

[89] Lovejoy KS, Lippard SJ. Non-traditional platinum compounds for improved accumulation, oral bioavailability, and tumor targeting. Dalton Trans 2009;28:10651–9. 10.1039/b913896j PMC2800312 20023892

[90] Zhang Z, Yu HJ, Wu S, et al. Synthesis, characterization, and photodynamic therapy activity of 5,10,15,20-Tetrakis. (Carboxyl)Porphyrin. Bioorg Med Chem 2019;27:2598–608. 30992204 10.1016/j.bmc.2019.03.051

[91] Zhou X, Tse MK, Wan TS, et al. Synthesis of beta-mono-, tetra-, and octasubstituted sterically bulky porphyrins via Suzuki Cross Coupling. J Org Chem 1996;31:3590–3. 10.1021/jo952205+ 11667202

[92] Huang Q, Pan ZQ, Wang P, et al. Zinc(II) and copper(II) complexes of beta-substituted hydroxylporphyrins as tumor photosensitizers. Bioorg Med Chem Lett 2006;16:3030–3. 16540316 10.1016/j.bmcl.2005.02.094

[93] Pan D, Zhong XM, Zhao WD, et al. Meso-substituted porphyrin photosensitizers with enhanced near-infrared absorption: synthesis, characterization and biological evaluation for photodynamic therapy. Tetrahedron 2018;74:2677–83.

[94] Pavani C, Uchoa AF, Oliveira CS, et al. Effect of zinc insertion and hydrophobicity on the membrane interactions and PDT activity of porphyrin photosensitizers. Photochem Photobiol Sci 2009;8:233–40. 19247516 10.1039/b810313e

[95] Zhu SZ, Wu FS, Wang K, et al. Photocytotoxicity, cellular uptake and subcellular localization of amidinophenylporphyrins as potential photodynamic therapeutic agents: an in vitro cell study. Bioorg Med Chem Lett 2015;25:4513–7. 26338364 10.1016/j.bmcl.2015.08.072

[96] Yu Q, Xu WX, Yao YH, et al. Synthesis and photodynamic activities of a new metronidazole-appended porphyrin and its Zn(II) complex. J Porphyrins Phthalocyanines 2015;19:1107–13.

[97] Brunner H, Schellerer KM, Treittinger B. Synthesis and in vitro testing of hematoporphyrin type ligands in platinum(II) complexes as potent cytostatic and phototoxic antitumor agents. Inorg Chim Acta 1997;264:67–79.

[98] Lottner C, Bart KC, Bernhardt G, Brunner H. Soluble tetraarylporphyrin-platinum conjugates as cytotoxic and phototoxic antitumor agents. J Med Chem 2002;45:2079–89. 11985475 10.1021/jm0110690

[99] Song R, Kim YS, Lee CO, et al. Synthesis and antitumor activity of DNA binding cationic porphyrin–platinum(II) complexes. Tetrahedron Lett 2003;44:1537–40.

[100] Brunner H, Gruber N. Carboplatin-containing porphyrin–platinum complexes as cytotoxic and phototoxic antitumor agents. Inorg Chim Acta 2004;357:4423–51.

[101] Naik A, Rubbiani R, Gasser G, Spingler B. Visible-light-induced annihilation of tumor cells with platinum-porphyrin conjugates. Angew Chem 2014;126:7058–61. 10.1002/anie.201400533 24852715

[102] Tasso TT, Tsubone TM, Baptista MS, et al. Isomeric effect on the properties of tetraplatinated porphyrins showing optimized phototoxicity for photodynamic therapy. Dalton Trans 2017;46:11037–45. 28787061 10.1039/c7dt01205e

[103] Hu X, Ogawa K, Li S, et al. A platinum functional porphyrin conjugate: an excellent cancer killer for photodynamic therapy. Bull Chem Soc Jpn 2019;92:790–6.

[104] Yang MQ, Deng JR, Guo D, et al. A folate-conjugated platinum porphyrin complex as a new cancer-targeting photosensitizer for photodynamic therapy. Org Biomol Chem 2019;17:5367–74. 31106316 10.1039/c9ob00698b

[105] Schmitt F, Govindaswamy P, Zava O, et al. Combined arene ruthenium porphyrins as chemotherapeutics and photosensitizers for cancer therapy. J Biol Inorg Chem 2009;14:101–9. 18810507 10.1007/s00775-008-0427-y

[106] Schmitt F, Govindaswamy P, Süss-Fink G, et al. Ruthenium porphyrin compounds for photodynamic therapy of cancer. J Med Chem 2008;51:1811–6. 18298056 10.1021/jm701382p

[107] Cunningham M, McCrate A, Nielsen M, Swavey S. Highly efficient visible-light-induced photocleavage of DNA by a ruthenium-substituted fluorinated porphyrin. Eur J Inorg Chem 2009;2009:1521–5.

[108] Schmitt F, Auzias M, Štěpnička P, et al. Sawhorse-type diruthenium tetracarbonyl complexes containing porphyrin-derived ligands as highly selective photosensitizers for female reproductive cancer cells. J Biol Inorg Chem 2009;14:693–701. 19241094 10.1007/s00775-009-0482-z

[109] Zhang JX, Wong KL, Wong WK, et al. Two-photon induced luminescence, singlet oxygen generation, cellular uptake and photocytotoxic properties of amphiphilic Ru(II) polypyridyl-porphyrin conjugates as potential bifunctional photodynamic therapeutic agents. Org Biomol Chem 2011;9:6004–10. 21748193 10.1039/c1ob05415e

[110] Pan J, Jiang L, Chan CF, et al. Excitation energy transfer in ruthenium (II)-porphyrin conjugates led to enhanced emission quantum yield and ^1^O_2_ generation. J Lumin 2017;184:89–95.

[111] Cabrera-González J, Soriano J, Conway-Kenny R, et al. Conway-Kenny R Multinuclear Ru(II) and Ir(III) decorated tetraphenylporphyrins as efficient PDT agents. Biomater Sci 2019;7:3287–96. 31187805 10.1039/c9bm00192a

[112] Barnard PJ, Berners-Price SJ. Targeting the mitochondrial cell death pathway with gold compounds. Coord Chem Rev 2007;251:1889–902.

[113] Milacic V, Dou QP. The tumor proteasome as a novel target for gold(III) complexes: implications for breast cancer therapy. Coord Chem Rev 2009;253:1649–60. 20047011 10.1016/j.ccr.2009.01.032 PMC2675785

[114] Ott I. On the medicinal chemistry of gold complexes as anticancer drugs. Coord Chem Rev 2009;253:1670–81.

[115] Che CM, Sun RW, Yu WY, et al. Gold(III) porphyrins as a new class of anticancer drugs: cytotoxicity, DNA binding and induction of apoptosis in human cervix epitheloid cancer cells. ChemCommun 2003;21:1718–9. 10.1039/b303294a 12877519

[116] Wang Y, He QY, Sun RW, et al. Cellular pharmacological properties of gold(III) porphyrin 1a, a potential anticancer drug lead. Eur J Pharmacol 2007;554:113–22. 17116302 10.1016/j.ejphar.2006.10.034

[117] Chen HS, Li J, Shen TT, et al. Gold(III) tetraarylporphyrin phosphonate derivatives as potential anticancer agents. J Chem Res 2012;36:501–5.

[118] Sun L, Chen HS, Zhang ZL, et al. Synthesis and cancer cell cytotoxicity of water-soluble gold(III) substituted tetraarylporphyrin. J Inorg Biochem 2012;108:47–52. 22265838 10.1016/j.jinorgbio.2011.12.003

[119] Lammer AD, Cook ME, Sessler JL. Synthesis and anti-cancer activities of a water soluble gold(III) porphyrin. J Porphyrins Phthalocyanines 2015;19:398–403. 10.1142/S1088424615500236 PMC4407279 25914517

[120] Longevial JF, Cheikh KEI, Aggad D, et al. Porphyrins conjugated with peripheral thiolato gold(I) complexes for enhanced photodynamic therapy. Chem Eur J 2017;23:14017–26. 28763126 10.1002/chem.201702975

[121] Hu X, Ogawa K, Kiwada T, Odani A. Water-soluble metalloporphyrinates with excellent photo-induced anticancer activity resulting from high tumor accumulation. J Inorg Biochem 2017;170:1–7. 28189031 10.1016/j.jinorgbio.2017.02.001

[122] Sour A, Jenni S, Orti-Suarez A, et al. Four gadolinium (III) complexes appended to a porphyrin: a water-soluble molecular theranostic agent with remarkable relaxivity suited for MRI tracking of the photosensitizer. Inorg Chem 2016;55:4545–54. 27074089 10.1021/acs.inorgchem.6b00381

[123] Chen YH, Zheng X, Dobhal MP, et al. Methyl pyropheophorbide-a analogues: potential fluorescent probes for the peripheral-type benzodiazepine receptor. Effect of central metal in photosensitizing efficacy. J Med Chem 2005;48:3692–5. 15916419 10.1021/jm050039k

[124] da Silva AR, Inada NM, Rettori D, et al. In vitro photodynamic activity of chloro(5,10,15,20-tetraphenylporphyrinato)indium(III) loaded-poly(lactide-co-glycolide) nanoparticles in LNCaP prostate tumour cells. J Photochem Photobiol B 2009;94:101–12. 19070504 10.1016/j.jphotobiol.2008.10.010

[125] Nakai M, Maeda T, Mashima T, et al. Syntheses and photodynamic properties of glucopyranoside-conjugated indium(III) porphyrins as a bifunctional agent. J Porphyrins Phthalocyanines 2013;17:1173–82.

[126] Mion G, Gianferrara T, Bergamo A, et al. Phototoxic activity and DNA interactions of water-soluble porphyrins and their rhenium(I) conjugates. ChemMedChem 2015;10:1901–14. 26332425 10.1002/cmdc.201500288

[127] Schneider R, Tirand L, Frochot C, et al. Recent improvements in the use of synthetic peptides for a selective photodynamic therapy. Anticancer Agents Med Chem 2006;6:469–88. 17017856 10.2174/187152006778226503

[128] Frochot C, Di Stasio B, Vanderesse R, et al. Interest of RGD-containing linear or cyclic peptide targeted tetraphenylchlorin as novel photosensitizers for selective photodynamic activity. Bioorg Chem 2007;35:205–20. 17223161 10.1016/j.bioorg.2006.11.005

[129] Srivatsan A, Ethirajan M, Pandey SK, et al. Conjugation of cRGD peptide to chlorophyll a based photosensitizer (HPPH) alters its pharmacokinetics with enhanced tumor-imaging and photosensitizing (PDT) efficacy. Mol Pharmaceutics 2011;8:1186–97. 10.1021/mp200018y PMC3148296 21702452

[130] Conway CL, Walker I, Bell A, et al. In vivo and in vitro characterisation of a protoporphyrin IX-cyclic RGD peptide conjugate for use in photodynamic therapy. Photochem Photobiol Sci 2008;7:290–8. 18389145 10.1039/b715141a

[131] Chaleix V, Sol V, Huang YM, et al. RGD-porphyrin conjugates: synthesis and potential application in photodynamic therapy. Eur J Org Chem 2003;2003:1486–93.

[132] Sibrian-Vazquez M, Jensen TJ, Fronczek FR, et al. Synthesis and characterization of positively charged porphyrin-peptide conjugates. Bioconjugate Chem 2005;16:852–63. 10.1021/bc050057g 16029027

[133] Chaloin L, Bigey P, Loup C, et al. Improvement of porphyrin cellular delivery and activity by conjugation to a carrier peptide. Bioconjugate Chem 2001;12:691–700. 10.1021/bc000125t 11562187

[134] Vives E. Cellular uptake of the Tat peptide: an endocytosis mechanism following ionic interactions. J Mol Recognit 2003;16:265–71. 14523939 10.1002/jmr.636

[135] Sibrian-Vazquez M, Jensen TJ, Hammer RP, et al. Syntheses and cellular studies of water soluble porphyrin-peptide conjugates. Proc Spie 2007;6427:64270A.

[136] Sibrian-Vazquez M, Jensen TJ, Hammer RP, Vicente M. Peptide-mediated cell transport of water soluble porphyrin conjugates. J Med Chem 2006;49:1364–72. 16480271 10.1021/jm050893b

[137] Sibrian-Vazquez M, Jensen TJ, Vicente M. Synthesis, characterization, and metabolic stability of porphyrin-peptide conjugates bearing bifunctional signaling sequences. J Med Chem 2008;51:2915–23. 18426194 10.1021/jm701050j

[138] Tréhin R, Merkle HP. Chances and pitfalls of cell penetrating peptides for cellular drug delivery. Eur J Pharm Biopharm 2004;58:209–23. 15296950 10.1016/j.ejpb.2004.02.018

[139] Sehgal I, Sibrian-Vazquez M, Vicente M. Photoinduced cytotoxicity and biodistribution of prostate cancer cell-targeted porphyrins. J Med Chem 2008;51:6014–20. 18839477 10.1021/jm800444c

[140] Sibrian-Vazquez M, Jensen TJ, Vicente M. Influence of the number and distribution of NLS peptides on the photosensitizing activity of multimeric porphyrin-NLS. Org Biomol Chem 2010;8:1160–72. 20165809 10.1039/b917280g

[141] Dondi R, Yaghini E, Tewari K, et al. Flexible synthesis of cationic peptide-porphyrin derivatives for light-triggered drug delivery and photodynamic therapy. Org Biomol Chem 2016;14:11488–501. 27886311 10.1039/c6ob02135b PMC5166568

[142] Abrahamse H, Kruger CA, Kadanyo S, Mishra A. Nanoparticles for advanced photodynamic therapy of cancer. Photomed Laser Surg 2017;35:581–8. 28937916 10.1089/pho.2017.4308

[143] Zeng JF, Yang WD, Shi DJ, et al. Porphyrin derivative conjugated with gold nanoparticles for dual-modality photodynamic and photothermal therapies in vitro. ACS Biomater Sci Eng 2018;4:963–72. 33418778 10.1021/acsbiomaterials.7b00886

[144] Xue YD, Tian J, Xu L, et al. Ultrasensitive redox-responsive porphyrin-based polymeric nanoparticles for enhanced photodynamic therapy. Eur Polym J 2019;110:344–54.

[145] Zhao TT, Wu H, Yao SQ, et al. Nanocomposites containing gold nanorods and porphyrin-doped mesoporous silica with dual capability of two-photon imaging and photosensitization. Langmuir 2010;26:14937–42. 20726559 10.1021/la102556u

[146] Penon O, Marín MJ, Amabilino DB, et al. Iron oxide nanoparticles functionalized with novel hydrophobic and hydrophilic porphyrins as potential agents for photodynamic therapy. J Colloid Interface Sci 2016;462:154–65. 26454374 10.1016/j.jcis.2015.09.060

[147] Wang D, Niu LJ, Qiao ZY, et al. Synthesis of self-assembled porphyrin nanoparticle photosensitizers. ACS Nano 2018;12:3796–803. 29611423 10.1021/acsnano.8b01010

[148] Bretin L, Pinon A, Bouramtane S, Ouk C, et al. Photodynamic therapy activity of new porphyrin-xylan-coated silica nanoparticles in human colorectal cancer. Cancers 2019;11:1474. 10.3390/cancers11101474 PMC6826978 31575052

[149] Pan D, Liang PP, Zhong XM, et al. Self-assembled porphyrin-based nanoparticles with enhanced near-infrared absorbance for fluorescence imaging and cancer photodynamic therapy. ACS Appl Bio Mater 2019;2:999–1005. 10.1021/acsabm.8b00530 35021390

[150] Frimayanti N, Yam ML, Lee HB, et al. Validation of quantitative structure-activity relationship (QSAR) model for photosensitizer activity prediction. Int J Mol Sci 2011;12:8626–44., 22272096 10.3390/ijms12128626 PMC3257093

[151] Siewert B, Stuppner H. The photoactivity of natural products – An overlooked potential of phytomedicines?. Phytomedicine 2019;60:152985. 31257117 10.1016/j.phymed.2019.152985

